# Does feeling collective responsibility for intergroup harm lead to infrahumanization?

**DOI:** 10.1371/journal.pone.0323292

**Published:** 2025-05-29

**Authors:** Robert A. Brennan, Florence E. Enock, Carl J. Bunce, Harriet Over

**Affiliations:** 1 Department of Psychology, University of York, York, United Kingdom; 2 The Institute of Future Media, Democracy and Society, Dublin City University, Dublin, Ireland; 3 The Alan Turing Institute, British Library, London, United Kingdom; 4 School of Psychology and Clinical Language Sciences, University of Reading, Reading, United Kingdom; The Open University of Israel, ISRAEL

## Abstract

Previous research has suggested that subtle dehumanization can occur as a consequence of harming others. According to this research, participants who feel a sense of collective responsibility for their ingroup contributing to the harm experienced by an outgroup may perceive members of that group as experiencing uniquely human emotions to a lesser extent, compared to when they do not feel responsible. We sought to understand whether these results replicate in two novel intergroup contexts: understanding UK residents’ perceptions of those who disproportionately experience the harmful effects of climate change in Nigeria, and employees working in exploitative conditions in fast fashion supply chains. Across three well-powered and pre-registered studies, we successfully manipulated perceived harm against the target groups and perceived ingroup responsibility for that harm. However, we found no evidence for the previously reported relationship between one’s sense of collective responsibility for harm and the perceived extent that target outgroup members experience uniquely human emotions. Instead, a more straightforward pattern was found in all three studies, whereby participants perceived outgroup members to experience negative emotions to a greater extent when harm towards them has been made salient. This trend was irrespective of whether the emotions were uniquely human (e.g., *admiration*, *shame*) or shared with other animals (e.g., *joy*, *sadness*). The often evoked relationship between the need to justify harm and the tendency to subtly dehumanize harmed outgroup members may be less common than is often assumed.

According to infrahumanization theory, a prominent emotion-based model of subtle dehumanization, outgroups are dehumanized to the extent that they are seen as lacking in uniquely human emotions compared to one’s ingroup [1,2]. Also known as secondary emotions, uniquely human emotions are considered complex and long-lasting, including nostalgia, pride, shame, and euphoria. In contrast, emotions shared with other animals (also known as primary emotions) are considered more primal and fleeting, representing experiences humans perceive as sharing with other animals, such as happiness, fear, sadness, and surprise. The distinction between uniquely human emotions and those we share with nonhuman animals is based on lay perceptions of human and animal emotion experiences, with some empirical evidence suggesting that people spontaneously categorize emotions along this dimension [3]. Empirical work has suggested that we infrahumanize many kinds of outgroups, including regional and national ones [4–6], immigrants [7–9], refugees [10,11], and members of minority ethnic groups [12–14]. Some individual factors have also been associated with a higher likelihood of outgroup infrahumanization occurring, such as holding conservative political attitudes [12,15].

One reason researchers across many disciplines are interested in the construct of dehumanization is that it is thought to be causally related to harm. Philosophers have noted that genocide is often preceded by propaganda in which the members of the target outgroup are explicitly described as rats, lice, and parasites [16,17]. Such blatant forms of dehumanization have also been linked to increased hostility in lab-based work. Kteily and Bruneau [18] reported that the extent to which US Americans endorse the claim that Arabs are “less than human” predicted hostility towards Arab immigration.

Subtle forms of dehumanization are also hypothesized to be causally related to harm [14,19–26]. For example, Cuddy et al. [27] asked participants to estimate the extent to which Hurricane Katrina survivors of their racial ingroup and outgroup experienced uniquely human emotions such as grief, sorrow, and mourning, as well as emotions shared with other animals such as anger, panic, and rage. The less their participants attributed uniquely human emotions to outgroup members, the less willing they were to offer them help. In related experimental work, Vaes et al. [28] found that participants responded more prosocially to a stranger who expressed a uniquely human emotion (*disappointment*) than to a stranger who expressed an emotion shared with other animals (*anger*).

Recently, the hypothesized causal relation between dehumanization and harm has been questioned [29–34]. Individuals and groups may often be harmed because of uniquely human qualities they are thought to possess. Perceiving a group to be enemies, criminals, or traitors, terms that make the most sense when applied to humans, might motivate harm or oppression towards members of that group [31–33,35,36]. Lab-based research has shown that the apparent causal relationship between infrahumanization and social behavior can often be explained by a confound in the stimuli used. It has been suggested that the uniquely human qualities incorporated into stimulus sets are often more prosocial than those shared with other animals [33,37]. When appropriate controls are put in place, lowered attributions of prosocial and socially desirable qualities predict a reduction in prosocial behavior and increases in antisocial behavior towards outgroup members, not lowered attributions of uniquely human qualities [37–42].

However, researchers have pointed out that dehumanization may be related to harm in other ways [43]. Perpetrators may justify the harm they have caused by coming to perceive their victims as less than human. The sociologist Luft [44] suggests that dehumanization might sometimes manifest as a result of violence rather than a predecessor to it. Based on research into cases of extreme intergroup violence, including the Rwandan genocide and the Holocaust, Luft argues that dehumanizing language may serve to reinforce social norms surrounding ongoing violence and justify the violence already undertaken by perpetrators. In particular, dehumanizing discourse may emerge to help alleviate the trauma and guilt that perpetrators feel in response to killing fellow humans [45]. In support of this analysis, perpetrators of extreme intergroup harm sometimes refer to individuals and groups they have harmed as less than human [16,46,47].

The hypothesis that dehumanization may occur as a consequence of, or response to, harm has also been investigated with more subtle forms of dehumanization in lab-based settings. In a seminal paper in this field, Castano and Giner-Sorolla [48] investigated whether participants would be more likely to deny outgroup members uniquely human emotions when they believed their ingroup was responsible for causing the outgroup harm. In one study, Castano and Giner-Sorolla presented participants with a scenario in which humans were either responsible for killing a large number of extra-terrestrial aliens or not. Participants were then asked to rate the extent to which they thought the aliens experienced 59 emotions on a 5-point Likert scale. Participants’ emotion attributions were correlated with previously reported data on the extent to which these emotions are perceived as unique to humans [49]. Results suggested that when participants felt responsible for their ingroup harming members of an outgroup, they attributed uniquely human emotions to them to a lesser extent than when they did not feel responsible. Castano and Giner-Sorolla [48] broadly replicated this effect of infrahumanization following harm in two further studies using real-world contexts. These post-harm contexts involved British participants rating the emotion typicality of Indigenous Australians, and European US Americans rating the emotion typicality of Native Americans.

Further evidence for a relationship between collective responsibility for harm and infrahumanization was provided by Čehajić et al. [50]. Serbian participants were asked to estimate the extent to which Bosnian Muslims experienced 16 emotions. In one condition, a sense of collective responsibility for the genocide of Bosniaks in the early 1990s was made salient, while in the other two conditions, it was not. For the emotion ratings, four emotions were unique to humans and positive to experience (*tenderness*, *hope*, *admiration*, and *love*), four were unique to humans and negative to experience (*remorse*, *guilt*, *shame*, and *resentment*), four were shared with other animals and positive to experience (*happiness*, *pleasure*, *euphoria*, and *joy*) and four were shared with other animals and negative to experience (*sadness*, *disgust*, *anger*, and *fear*). Participants rated Bosnian Muslims as typically feeling uniquely human emotions to a lesser extent when ingroup responsibility for harm was made salient compared to when it was not. These results were replicated in another context by Čehajić and colleagues, whereby European Chileans rated Indigenous Chileans as experiencing uniquely human emotions to a lesser extent when encouraged to feel responsible for the harm European colonialism brought upon the Native peoples of Chile. Overall, the studies described by Castano and Giner-Sorolla [48] and Čehajić et al. [50] reflect an apparent motivation to alleviate negative affect in response to harming fellow human beings by attributing fewer uniquely human emotions to them.

The research offering evidence of infrahumanization following harm are not without limitations. For instance, the crucial interaction between group condition and emotion humanness did not always reach significance, such as in Study 1 in Castano and Giner-Sorolla [48] and Studies 1 and 2 in Čehajić et al. [50]. However, the researchers broke down these nonsignificant interactions and treated the trends found within as evidence for infrahumanization following harm. Each piece of research also had sampling limitations. Castano and Giner-Sorolla [48] recruited relatively small samples of psychology undergraduates in their first two studies, as did Čehajić et al. [50] in their first study. The sample collected by Čehajić and colleagues for their Study 2 comprised teenage students at a specific secondary school. The combination of marginally significant trends and small samples in the two most influential papers that claim to show evidence of infrahumanization following harm warrant an examination of how widely applicable these findings are to other contexts.

Considering the above concerns, as well as recent theoretical and empirical critiques of infrahumanization theory and its role in intergroup harm more broadly, we sought to measure whether we could conceptually replicate the occurrence of infrahumanization in response to harm in two contemporary contexts – the harm caused to people by climate change and the harm caused to people by the fast fashion industry. Across three well-powered and pre-registered studies, we sought to test whether the previously reported relationship between feeling collective responsibility for harming an outgroup and the infrahumanization of its members replicates in these two intergroup contexts.

Following previous research, we compare participants’ performance across three conditions. In the *harm responsible condition*, we inform participants that the outgroup has experienced harm and that the ingroup is responsible for causing that harm. We compare emotion attribution in this condition to two other conditions. In the *harm not responsible condition*, we informed participants that the outgroup had experienced the same harm but that responsibility for the harm fell on a third party unrelated to the participants’ ingroup. In the control *no harm condition*, we describe members of the same outgroup without any mention of harm. Regarding the predictions of previous research employing infrahumanization theory [48,50], the crucial comparison is between the two harm conditions. If infrahumanization occurs as a response to collective harm, then participants should attribute uniquely human emotions to members of the outgroup more strongly in the harm not responsible condition than in the harm responsible condition.

## Methods

### Ethical review and open science

All studies received ethical approval from the Psychology Departmental Ethics Committee at the University of York (approval number 127). All data collection occurred online, and the studies were created and administered using Qualtrics (https://www.qualtrics.com). Participants were recruited through the online platform Prolific (https://www.prolific.co), with an independent sample recruited for each study. Informed consent was obtained through an online form for all studies. Participants were provided with an information sheet detailing the study’s purpose, their rights, and any potential risks. They indicated their understanding and consent by ticking a checkbox, confirming they had read the information sheet, understood their rights, and agreed to participate. Only individuals who provided this electronic consent were able to progress to the study. Participants were rewarded at an approximate rate of £9 per hour in all studies.

Assumption testing and subsequent frequentist analyses were conducted using *SPSS* and *RStudio*. Violation of the assumptions of normality in Studies 1 & 2, and homogeneity of variance in Study 2 were not considered a major concern as the mixed factorial design with repeated measures, paired with an equal, large number of participants in these studies provide a sufficiently robust design [51,52]. As can be seen in the analysis Rscript, alternative designs further confirmed these violated assumptions did not affect the results. All other assumptions were met for all studies. Bayesian analyses were conducted in JASP using default Cauchy prior distributions to quantify evidence for null hypotheses. Bayes factors (BF₀₁) above 1, 3 and 10 are typically interpreted as anecdotal, moderate and strong support for the null, respectively. All post hoc tests were Bonferroni corrected for multiple comparisons. Highly influential cases were identified using Cook’s distance in each study. All Studies were pre-registered on AsPredicted.com before commencing data collection. Data files [53], pre-registration documents, the Rscript for main analyses, equivalence testing, and the stimuli used for each study can be seen in the online supplementary materials available on OSF at https://doi.org/10.17605/OSF.IO/53WHS.

## Study 1: Testing for infrahumanization of outgroup members harmed by climate change

In Study 1, we examined whether UK residents perceived residents of Lagos, Nigeria, as typically experiencing uniquely human emotions to a lesser extent when they felt collective responsibility for the harm they experience due to climate change, following the same design as Čehajić and colleagues [50]. This intergroup context was chosen because Lagos is a region considered particularly vulnerable to the consequences of climate change, with locals already experiencing some harmful effects [54,55].

We asked participants to read paragraphs about the residents of Lagos and then rate the extent to which residents of Lagos typically experienced the same selection of 16 different emotions used by Čehajić and colleagues [50]. The content of the paragraphs differed between the three conditions. In the harm responsible condition, the responsibility of UK residents for the harm experienced by the residents of Lagos due to climate change was emphasized. In the harm not responsible condition, the responsibility of multinational oil companies for this harm was emphasized. In the no harm condition, participants read a paragraph about residents of Lagos in which no harm or effects of climate change were mentioned.

We sought to measure whether participants would rate residents of Lagos as typically experiencing uniquely human emotions to a lesser extent when participants felt a sense of collective responsibility for the harm caused to the target group by climate change, compared to when participants did not feel responsible for that harm or when no harm was emphasized.

## Method

### Participants

A power analysis using G*Power indicated that a sample size of 249 would allow us to detect a medium effect size (*η*_p_² = .06) with an alpha of .05 and power of .95 using a mixed ANOVA. A final sample of 252 participants was included to allow for an equal number of 84 participants between condition groups. Online recruitment for this study occurred on July 14^th^, 2022. All participants were adult UK nationals currently residing in the UK who were fluent in English and had an approval rating of at least 95% on Prolific. Data from two participants were omitted and replaced as they failed attention checks. Data from two further participants were excluded as they were mistakenly recruited after the pre-registered sample size of 252 participants had been met. Including data submitted by excess participants in analyses did not change the results. Participants’ ages ranged from 18 to 79 (*M = *41.3, *SD* = 15.9), and 158 identified as female, 92 as male and two as nonbinary. As holding conservative political attitudes is a known demographic predictor of infrahumanization [12,15], counterbalancing during recruitment ensured the sample was balanced regarding participants’ political orientation, with 127 indicating they were left-leaning and 125 right-leaning. Differences in political orientation did not influence the key effects of interest, with a moderated model yielding the same results as those reported.

## Materials

**Vignettes.** There were three between-subject conditions: *Harm responsible*, *Harm not responsible* and *No harm*. In the two harm conditions, participants first read the same four paragraphs detailing the extent to which climate change has harmed the residents of Lagos, Nigeria. Those in the harm responsible condition then read further paragraphs detailing the contribution of UK residents to climate change. In contrast, those in the harm not responsible condition read further paragraphs detailing the contribution of multinational oil companies. Participants in the no harm condition read paragraphs describing life for the residents of Lagos without mentioning climate change or harm. The vignettes can be seen in the online supplementary materials.

**Manipulation checks.** All participants completed the same two manipulation checks. Participants were first asked, *“How harmful do you think climate change is to the residents of Lagos?*”. This manipulation check appeared after the paragraphs describing Lagos, with or without mentioning the harmful effects of climate change in the region. It was included to ensure that participants in the two harm conditions perceived the residents of Lagos as experiencing more harm than participants in the no harm condition.

The second manipulation check asked, “*How responsible do you think UK citizens are for the effects of climate change on residents of Lagos?”* which appeared after the second set of paragraphs for participants in the two harm conditions. This check was included to ensure that a greater sense of collective responsibility for the harm experienced by the residents of Lagos due to climate change was felt by participants in the harm responsible condition than those in the harm not responsible condition. Participants in the no harm condition also responded to this manipulation check, which was presented alongside the *perceived harm* manipulation check once they read the single set of paragraphs in this condition.

Both manipulation checks were responded to using unmarked sliding scales ranging from 0 (*Not at all*) to 100 (*Very much so*), with the sliders initially fixed at the midpoint of the scale (*Somewhat*).

**Outgroup emotion typicality ratings.** Following the between-subjects experimental manipulation, all participants rated the extent to which residents of Lagos typically experience 16 different emotions. The selection of emotions was the same as used by Čehajić et al. [50], consisting of four uniquely human emotions that are positive to experience (*tenderness, hope, admiration, love*), four uniquely human emotions that are negative to experience (*remorse, guilt, shame, resentment*), four emotions shared with other animals that are positive to experience (*happiness, euphoria, pleasure, joy*), and four emotions shared with other animals that are negative to experience (*sadness, disgust, anger, fear*). We chose the emotions used by Čehajić et al. [50] because their design closely mirrors that used in many other studies of infrahumanization [1,2,6,56,57] and because personal communication with Castano and Giner-Sorolla [48] revealed these researchers no longer had access to the specific list of stimuli used in their previous work. Participants responded to the extent to which they believed the outgroup to experience each item using an unmarked sliding scale ranging from 0 (*Not at all*) to 100 (*Very much so*), with the sliders initially fixed at the midpoint of the scale (‘*Somewhat’*). The 16 emotion items were presented in a randomized order. An attention check (*Residents typically: please indicate ‘not at all*’) appeared approximately midway through this scale.

## Design

This study had a 3 (Condition: harm responsible, harm not responsible, and no harm; between) X 2 (Emotion humanness: uniquely human and shared with other animals; within) X 2 (Emotion valence: positive and negative; within) mixed design. Data were analyzed using a mixed ANOVA for the main analysis.

## Procedure

Interested individuals were informed on Prolific that the study aimed to examine how people ascribe emotions to different groups of individuals. After providing informed consent, participants answered brief demographic questions, including gender identity, age, political orientation, and checks for the inclusion criteria. Participants then proceeded to the experimental stimuli.

Participants in the two harm conditions read four paragraphs describing harm to residents of Lagos due to climate change and then responded to the first manipulation check measuring their perceived extent of harm. Participants in these two harm conditions then read four more paragraphs intended to manipulate their sense of collective responsibility for the harm. Following this, participants responded to the second manipulation check, measuring participants’ sense of collective responsibility for the harm.

Participants in the no harm condition read four paragraphs describing aspects of life for residents of Lagos without mentioning climate change or responsibility for harm. These participants then responded to the same two manipulation checks one after another. Each paragraph was presented for at least eight seconds (without an upper limit) to ensure participants read each section carefully.

After reading the respective paragraphs for the experimental manipulation, participants in all three conditions responded to the outgroup emotion typicality items. Following this, participants could provide optional feedback on the study and were debriefed before returning to Prolific to complete the submission and receive payment. The study took an average of five and a half minutes to complete.

## Results

### Manipulation checks

**Fig 1:**
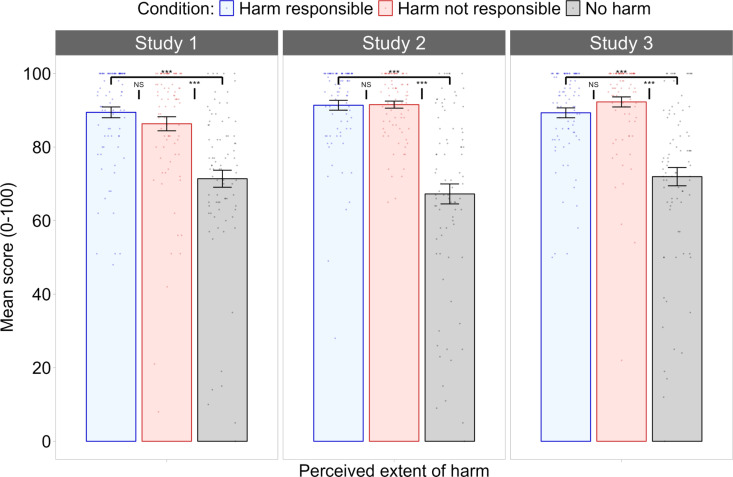
Mean scores for perceived extent of harm in Studies 1, 2, and 3. Perceived extent of harm was manipulated as intended in Study 1 (left panel), Study 2 (middle panel), and Study 3 (right panel). Participants in the harm responsible and harm not responsible conditions perceived outgroup members as experiencing significantly more harm than did participants in the no harm condition. *Note:* Error bars represent ±1 *SE*. *** denotes *p *< .001; *NS* denotes non-significant.

**The Perceived Extent of Harm.** A one-way between-subjects ANOVA revealed a significant difference in the perceived extent of harm experienced by residents of Lagos as a result of climate change, *F*(2, 249) = 25.09, *p* < .001, *η*² = .17, BF_01_ < 0.01. Post hoc comparisons indicated no significant difference between the harm responsible (*M* = 89.4, *SE* = 1.93) and the harm not responsible conditions (*M* = 86.3, *SE = *1.93), *p* = .77, 95% CI [-3.46, 9.68] BF_01_ = 2.78. The perceived extent of harm was significantly lower in the no harm condition (*M* = 71.4, *SE = *1.93) than in the harm responsible condition, *p* < .001, 95% CI [−24.63, −11.49], BF_01_ < 0.01and the harm not responsible condition, *p* < .001, 95% CI [−21.52, −8.38], BF_01_ < 0.01. The perceived extent of harm was thus manipulated as intended between conditions in Study 1, as illustrated in the left panel of Fig 1.

**Fig 2:**
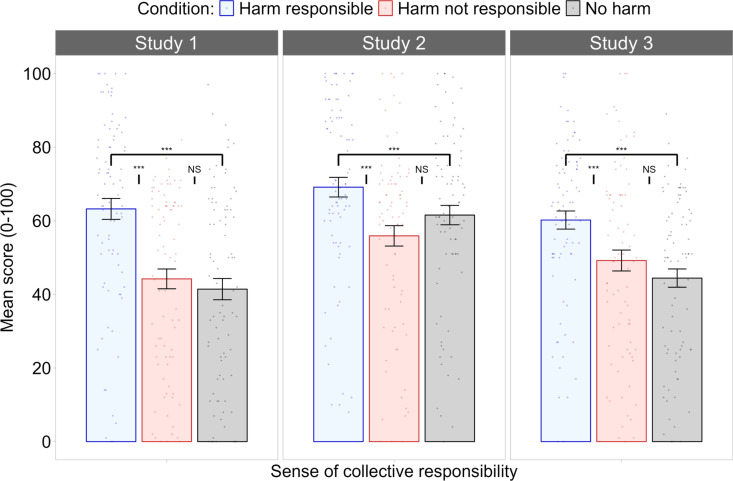
Mean scores for sense of collective responsibility in Studies 1, 2, and 3. Sense of collective responsibility in Study 1 (left panel), Study 2 (middle panel), and Study 3 (right panel). Participants in the harm responsible condition felt that their ingroup was significantly more responsible for the harm experienced by outgroup members than did participants in the harm not responsible condition. *Note:* Error bars represent ±1 *SE*. *** denotes *p *< .001; ** denotes *p* < .005; * denotes *p* < .05; *NS* denotes non-significant.

**Sense of Collective Responsibility.** A one-way between-subjects ANOVA revealed a significant difference between conditions in the extent to which participants felt a sense of collective responsibility for the harm caused by climate change, *F*(2, 249) = 17.84, *p* < .001, *η²* = .13, BF_01_ < 0.01. Post hoc comparisons revealed that participants in the harm responsible condition felt significantly more collective responsibility for the harm (*M* = 63.2, *SE* = 2.81) than did those in the harm not responsible condition (*M* = 44.2, *SE* = 2.81), *p* < .001, 95% CI [9.44, 28.59], BF_01_ < 0.01, or those in the no harm condition (*M* = 41.4, *SE* = 2.81), *p* < .001, 95% CI [12.22, 31.37], BF_01_ < 0.01. There was no significant difference in collective responsibility between the harm not responsible and no harm conditions, *p = *1, 95% CI [−6.79, 12.36], BF_01_ = 4.76. Therefore, this variable was also manipulated as intended between conditions, as seen in the left panel of Fig 2.

### Emotion ratings

**Fig 3:**
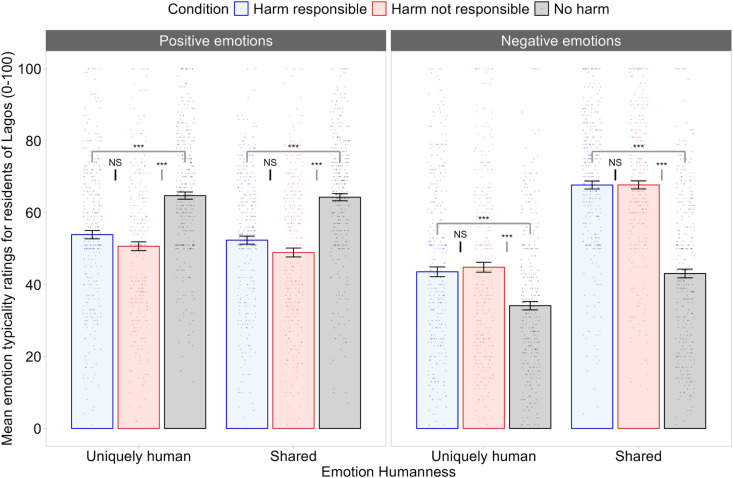
Results of Study 1. Mean emotion attributions by harm condition, emotion humanness, and emotion valence in Study 1 are shown. We observed no evidence that collective responsibility for harm induced infrahumanization of the outgroup. Instead, residents of Lagos were thought to experience negative emotions to a greater extent and positive emotions to a lesser extent when harm to them was made salient. *Note*: Error bars represent ±1 *SE*. Shared = emotions shared with other animals. *** denotes *p *< .001; *NS* denotes non-significant.

We conducted a 3 (Condition: harm responsible, harm not responsible, no harm; between) X 2 (emotion humanness: uniquely human, shared with other animals; within) X 2 (emotion valence: positive, negative; within) mixed ANOVA to examine if the target group were perceived as experiencing uniquely human emotions to a lesser extent when ingroup collective responsibility for harm was made salient compared to when ingroup responsibility was not made salient or no harm against the target group was described. The results are illustrated in Fig 3.

We found significant two-way interactions between harm condition and emotion humanness *F*(2, 249) = 20.270, *p* < .001, *η*_*p*_*² = *.14, BF_01_ < 0.01, and between harm condition and emotion valence, *F*(2, 246) = 57.714, *p* < .001, *η*_*p*_² = .32, BF_01_ < 0.01. The two-way interactions were each qualified by a significant three-way interaction between harm condition, emotion humanness, and emotion valence, *F*(2, 249) = 19.209, *p* < .001, *η*_*p*_² = .13, BF_01_ < 0.01. This three-way interaction was broken down using Bonferroni corrected pairwise comparisons between the three conditions at each level of emotion humanness and emotion valence.

**Positive Uniquely Human Emotions.** The extent to which residents of Lagos were thought to experience positive uniquely human emotions did not differ between the harm responsible condition (*M = *53.9, *SE* = 1.61) and the harm not responsible condition (*M = *50.7, *SE = *1.61), *p* = .475, 95% CI [-2.27, 8.71], BF_01_ < 0.01. Residents of Lagos were seen as typically experiencing positive uniquely human emotions to a greater extent in the no harm condition (*M = *64.8, *SE = *1.61) than in either the harm responsible condition, *p* < .001, 95% CI [5.36, 16.34], BF_01_ < 0.01, or the harm not responsible condition, *p* < .001, 95% CI [8.58, 19.57], BF_01_ < 0.01.

**Negative Uniquely Human Emotions**. The extent to which residents of Lagos were thought to experience negative uniquely human emotions did not differ between the harm responsible (*M* = 43.6, *SE* = 1.66) and harm not responsible (*M* = 44.8, *SE* = 1.66) conditions, *p* = 1, 95% CI [-6.95, 4.39], BF_01_ = 5.10. Residents of Lagos were seen as typically experiencing negative uniquely human emotions to a lesser extent in the no harm condition (*M* = 34.1, *SE* = 1.66) than in the harm responsible condition, *p* < .001, 95% CI [-15.09, -3.75], BF_01_ < 0.01, or in the harm not responsible condition, *p* < .001, 95% CI [-16.37, -5.03], BF_01_ < 0.01.

**Positive Shared Emotions.** A similar pattern emerged for positive emotions shared with other animals as with positive uniquely human emotions. There was no significant difference in the perceptions of the experience of positive shared emotions between the harm responsible condition (*M = *52.4, *SE* = 1.82) and the harm not responsible condition (*M = *48.9, *SE = *1.82), *p* = .545, 95% CI [-2.75, 9.63], BF_01_ = 2.96. Participants rated residents of Lagos as typically experiencing positive emotions shared with other animals to a greater extent in the no harm condition (*M = *64.3, *SE = *1.82) than in the harm responsible condition, *p* < .001, 95% CI [5.75, 18.13], BF_01_ < 0.01, or in the harm not responsible condition, *p* < .001, 95% CI [9.12, 21.57], BF_01_ < 0.01.

**Negative Shared Emotions.** A similar pattern emerged for negative emotions shared with other animals and negative uniquely human emotions. No significant difference in ratings of negative shared emotions was found between the harm responsible condition (*M* = 67.7, *SE* = 1.85) and the harm not responsible condition (*M* = 67.7, *SE* = 1.85), *p* = 1, 95% CI [-6.32, 6.26], BF_01_ = 6.00. Participants rated residents of Lagos as typically experiencing negative emotions shared with other animals to a lesser extent in the no harm condition (*M* = 43.1, *SE* = 1.85) than in the harm responsible condition, *p* < .001, 95% CI [-30.89, -18.31], BF_01_ < 0.01, or in the harm not responsible condition, *p *< .001, 95% CI [-30.92, -18.34], BF_01_ < 0.01.

## Study 1 Discussion

Our pattern of results does not align with previous research suggesting that a sense of collective responsibility for harm leads to the infrahumanization of the victim group, whereby individuals subtly dehumanize members of an outgroup they believe their ingroup has harmed [48,50]. Rather, our data show a somewhat more straightforward pattern. Participants estimated that the residents of Lagos were likely to feel negative emotions to a greater extent and positive emotions to a lesser extent when harm to this group was made salient, regardless of the humanness of the emotions and regardless of ingroup responsibility for this harm. This pattern is in striking contrast to previous findings.

While we did not replicate the central finding of previous research, our manipulation checks showed that we successfully manipulated perceived harm and perceived collective responsibility for that harm. These successful manipulations suggest that we should have been able to detect infrahumanization effects if they occur in this context.

## Study 2: Testing for infrahumanization of outgroup members harmed by the fast fashion industry

In Study 2, we sought to test whether collective responsibility for harm induced a tendency to infrahumanize the outgroup in another contemporary intergroup context. In this study, we examined whether UK residents rated textile workers in fast fashion supply chains as experiencing uniquely human emotions to a lesser extent when ingroup responsibility for harm was made salient compared to when it was not.

We chose this intergroup context for several reasons. The exploitation of textile workers is often referred to as dehumanizing [58,59]. Importantly, and like the climate change context used in Study 1, the fast fashion context provides the opportunity to frame the participant ingroup (UK residents) as either responsible or not responsible for the harm experienced by outgroup members. As in Study 1, this contemporary context of intergroup harm, where a participant’s actions as a member of a privileged group are directly contributing to the harm experienced by members of a disadvantaged outgroup, is arguably more relatable for participants than historical cases of harm, or entirely hypothetical ones, as previous research has examined [48,50].

As in Study 1, UK residents participated in one of three between-subject conditions. In the two harm conditions, participants read a series of paragraphs about the extent to which textile workers experience exploitation in fast fashion supply chains. In the harm responsible condition, participants then read about the high rates of fast fashion consumption in the UK. In the harm not responsible condition, participants read about the negligence of CEOs and boards of directors in fast fashion companies who prioritize profit over safe and fair working conditions for textile workers. In the no harm condition, participants read a few paragraphs detailing the history and global presence of the fast fashion industry without mentioning the harm textile workers experience. If people tend to infrahumanize others when they feel responsible for harm against them (as is predicted by past work in infrahumanization theory), we would expect to see greater attribution of uniquely human emotions in the harm not responsible and no harm conditions than in the harm responsible condition. We included the same emotion items as in Study 1, the same selection used by Čehajić and colleagues [50].

## Method

### Participants

The sample size estimation was the same as in Study 1. A sample of 252 participants was collected, with online recruitment for this study occurring on August 15^th^, 2022. All participants were adult UK nationals currently residing in the UK who were fluent in English and had an approval rating of at least 95% on Prolific. In the sample, 154 participants identified as female, 95 as male, two as nonbinary and one as unspecified. Ages ranged from 18 to 77 (*M = *41.1, *SD* = 15.6). We sought to recruit participants across the political spectrum, with 127 identifying as left-leaning and 125 as right-leaning. As in Study 1, differences in political orientation did not influence the key effects of interest, with a moderated model yielding the same results as those reported.

### Materials

The manipulation checks measuring the perceived extent of harm and sense of collective responsibility, the 16-item outgroup emotion typicality scale and attention checks were the same as in Study 1, with the target outgroup and contextual details adapted to the fast fashion context.

**Vignettes.** As in Study 1, the stimuli varied between the three between-subject conditions, with data from 84 participants collected for each (*harm responsible*, *harm not responsible* and *no harm*). In the two harm conditions, participants read four paragraphs detailing the extent of harm that poor working conditions and exploitation have on textile workers along fast fashion supply chains. After these four paragraphs, those in the harm responsible condition read four paragraphs describing the growing demand for fast fashion products UK consumers have and how this is complicit with the harm experienced by textile workers, allowing it to continue. Participants in the harm not responsible condition instead read four paragraphs framing fast fashion companies’ CEOs and boards of directors as responsible for the harm due to their prioritizing profits over safe and fair working conditions for textile workers in the supply chains. Participants in the no harm condition read four paragraphs detailing the history of fast fashion and the number of textile workers the fast fashion industry employs. In this vignette, there was no mention of harm.

### Procedure and design

The procedure and design in Study 2 were identical to those used in Study 1.

## Results

### Manipulation checks

**The Perceived Extent of Harm.** A one-way between-subjects ANOVA revealed a significant difference between the three conditions in the perceived extent of harm experienced by textile workers in fast fashion supply chains, *F*(2, 249) = 57.99, *p* < .001, *η*² = .32, BF_01_ < 0.01. Bonferroni-corrected post hoc comparisons revealed no significant difference in perceived harm between the harm responsible (*M* = 91.4, *SE* = 1.84) and harm not responsible conditions (*M* = 91.5, *SE* = 1.84), *p* = 1, 95% CI [-6.42, 6.09], BF_01_ = 5.97. The perceived extent of harm was significantly lower among participants in the no harm condition (*M* = 67.3, *SE* = 1.84) than those in the harm responsible condition, *p* < .001, 95% CI [-30.37, -17.86], BF_01_ < 0.01, or the harm not responsible condition, *p* < .001, 95% CI [-30.54, -18.03], BF_01_ < 0.01. Mean scores for participants’ perceived extent of harm in Study 2 are illustrated in the middle panel of Fig 1.

**Sense of Collective Responsibility.** A one-way between-subjects ANOVA revealed that the collective responsibility felt by participants significantly differed between conditions, *F*(2, 249) = 6.089, *p* = .003, *η*² = .05, BF_01_ < 0.01. Bonferroni-corrected post hoc comparisons revealed that participants in the harm responsible condition felt significantly more collective responsibility (*M* = 69.1, *SE* = 2.69) than those in the harm not responsible condition (*M* = 55.9, *SE = *2.69), *p* = .002, 95% CI [4.06, 22.39], BF_01_ = 0.03. In this study, no significant differences in collective responsibility were found between the no harm condition (*M = *61.6, *SE = *2.69) and either the harm responsible condition, *p* = .142, 95% CI [-16.75, 1.58], BF_01_ = 0.91, or the harm not responsible condition, *p* = .418, 95% CI [-3.53, 14.81], BF_01_ = 2.19. Mean scores for participants’ sense of collective responsibility in Study 2 are illustrated in the middle panel of Fig 2.

### Emotion ratings

We conducted a 3 (condition: harm responsible, harm not responsible, no harm; between) X 2 (emotion humanness: uniquely human, shared with other animals, within) X 2 (emotion valence: positive, negative, within) mixed ANOVA to examine if collective responsibility for harm influenced the extent to which participants perceived textile workers in fast fashion supply chains as typically experiencing uniquely human emotions and positive emotions. The main effects and a breakdown of the two-way interactions are reported in the online supplementary materials.

**Fig 4:**
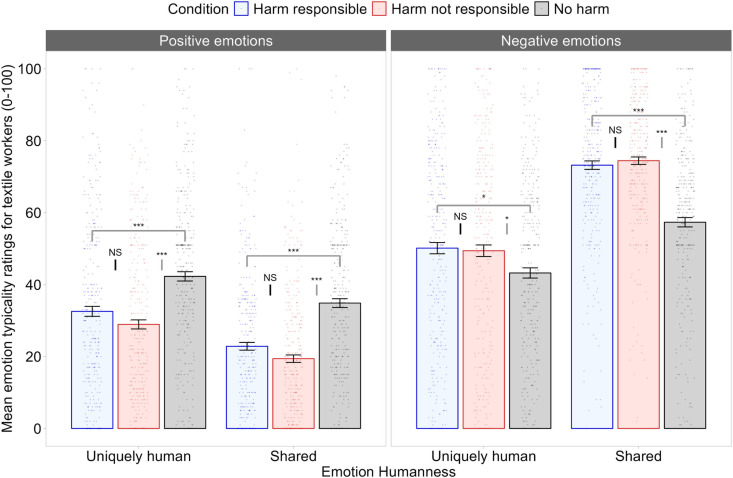
Results of Study 2. Mean emotion attributions by condition, emotion humanness, and emotion valence are displayed. We found no evidence that inducing a sense of collective responsibility for the harm experienced by textile workers leads participants to infrahumanize them. Converging with the results of Study 1, participants seemed to rate outgroup members as experiencing negative emotions more strongly when harm against them was made salient. *Note*: Error bars represent ±1 *SE*. *Shared* = emotions shared with other animals. *** denotes *p *< .001; * denotes *p *< .05; *NS* denotes non-significant.

The results of Study 2 are illustrated in Fig 4. We found significant two-way interactions between harm condition and emotion humanness, *F*(2, 249) = 5.647, *p* = .004, *η*_*p*_*² = *.04, BF_01_ < 0.01, and between harm condition and emotion valence, *F*(2, 246) = 29.730, *p* < .001, *η*_*p*_² = .19, BF_01_ < 0.01. As in Study 1, these were qualified by a significant three-way interaction between harm condition, emotion humanness, and emotion valence, *F*(2, 249) = 10.170, *p* < .001, *η*_*p*_² = .08, BF_01_ < 0.01. This three-way interaction was broken down using Bonferroni-corrected pairwise comparisons between the three conditions at each level of emotion humanness and emotion valence.

**Positive Uniquely Human Emotions.** The extent to which textile workers were thought to experience positive uniquely human emotions did not differ between the harm responsible condition (*M = *32.6, *SE* = 2.00) and the harm not responsible condition (*M = *28.9, *SE = *2.00), *p* = .601, 95% CI [-3.18, 10.44], BF_01_ = 2.67. Textile workers were seen as typically experiencing positive uniquely human emotions to a greater extent in the no harm condition (*M = *42.30, *SE = *2.00) than in the harm responsible condition *p* = .002, 95% CI [2.92, 16.54], BF_01_ = 0.04 or in the harm not responsible condition, *p* < .001, 95% CI [6.55, 16.54], BF_01_ < 0.01.

**Negative Uniquely Human Emotions**. The extent to which textile workers were thought to experience negative uniquely human emotions did not differ between the harm responsible (*M* = 50.2, *SE* = 1.74) and harm not responsible (*M* = 49.4, *SE = *1.74) conditions, *p* = 1, 95% CI [-5.19, 6.64], BF_01_ = 5.74. Textile workers were seen as typically experiencing negative uniquely human emotions to a lesser extent in the no harm condition (*M* = 43.2, *SE = *1.74) than in either the harm responsible condition *p* = .015, 95% CI [-12.82, -0.99], BF_01_ = 0.17, or the harm not responsible condition, *p *= .037, 95% CI [-12.10, -0.27], BF_01_ = 0.37.

**Positive Shared Emotions.** A similar pattern emerged for positive emotions shared with other animals as with positive uniquely human emotions. There was no significant difference in the perceptions of textile workers’ experiences of positive shared emotions between the harm responsible condition (*M = *22.9, *SE* = 1.91) and the harm not responsible condition (*M = *19.4, *SE = *1.91), *p* = .606, 95% CI [-3.05, 9.95], BF_01_ = 2.50. Participants rated textile workers as typically experiencing positive emotions shared with other animals to a greater extent in the no harm condition (*M = *34.9, *SE = *1.91) than in the harm responsible condition, *p* < .001, 95% CI [5.51, 18.51], BF_01_ < 0.01, or in the harm not responsible condition, *p* < .001, 95% CI [8.96, 21.96], BF_01_ < 0.01.

**Negative Shared Emotions.** A similar pattern emerged for negative emotions shared with other animals and negative uniquely human emotions. No significant difference in ratings of negative shared emotions was found between harm responsible condition (*M* = 73.2, *SE* = 1.77) and the harm not responsible condition (*M* = 74.5, *SE* = 1.77), *p* = 1, 95% CI [-7.26, 4.77], BF_01_ = 5.17. Participants rated textile workers as typically experiencing negative emotions shared with other animals to a lesser extent in the no harm condition (*M* = 57.4, *SE* = 1.77) than in the harm responsible condition, *p* < .001, 95% CI [-21.89, -9.86], BF_01_ < 0.01, or in the harm not responsible condition, *p* < .001, 95% CI [-23.13–11.10], BF_01_ < 0.01.

## Study 2 Discussion

Using the context of harm inflicted on fast fashion textile workers, we did not replicate the previously reported relationship between feeling collective responsibility for harm and infrahumanization. However, we observed a pattern of results that was very similar to that in Study 1. When harm was made salient, workers in fast fashion supply chains were thought to experience negative emotions to a greater extent and positive emotions to a lesser extent, regardless of emotion humanness or ingroup responsibility.

There are several possible reasons for our failure to conceptually replicate previous findings. One possibility is that we happened to choose intergroup contexts in which infrahumanization does not occur. Previous research has examined the relationship between collective responsibility for harm and infrahumanization in response to hypothetical scenarios with aliens as well as in real-world contexts such as responses to atrocities committed during colonialism and genocide [48,50]. We chose more contemporary and relatable intergroup contexts, focusing on the responsibility of UK residents for harm towards people affected by climate change and fast fashion textile workers. However, we successfully manipulated perceived harm and, when comparing the harm responsible and harm not responsible conditions, a sense of collective responsibility for harm, suggesting these are contexts where we would likely observe infrahumanization following harm if it occurs. Nevertheless, it remains possible that collective responsibility induces infrahumanization in some contexts but not others.

Another possibility is that the design we chose was insensitive to the effects of infrahumanization. Our design broadly mirrored that of Čehajić et al. [50]; however, infrahumanization research has been criticized more broadly for using a small number of emotion terms in each condition [42,60]. Castano and Giner-Sorolla [48] asked participants to rate a larger number of emotion terms that vary continuously along the dimensions of humanness and valence. In Study 3, we returned to the intergroup context of climate change used in Study 1. However, we sought to test the relationship between collective responsibility and infrahumanization with a more sensitive design similar to that used by Castano and Giner-Sorolla [48].

## Study 3: Testing for infrahumanization of outgroup members harmed by climate change using continuous measures

In Study 3, we again manipulated perceptions of harm inflicted on residents of Lagos caused by climate change and collective responsibility for this harm. This time, rather than measuring infrahumanization using a small number of emotions clustered into discrete categories, we asked participants to rate how typically the outgroup members experience a much larger range of emotions (64 in total). Before the manipulation, we asked participants to rate these emotions on the dimensions of humanness and valence. Asking the same participants to rate emotions prior to the outgroup measures might help to avoid inconsistencies in how human or valenced an emotion is considered to be between our sample and another. For instance, should we have used pre-test data, some emotions might be seen as more human than in the experimental sample we recruit. Such inconstancies can be seen in previous work, such as *disgust* being included in some study designs as an emotion shared with other animals [3,61] but in other studies as a uniquely human emotion [12,62]. Our initial intention was to use the same set of emotions used by Castano and Giner-Sorolla [48]. However, personal communication with the authors revealed no record of the exact stimuli they used. Instead, we selected 64 emotion terms based on previous research in the area, including Demoulin et al. [49] and Enock et al. [39].

In this design, infrahumanization is said to be reflected in a negative relationship between ratings of emotion humanness and typicality of outgroup members such that the more uniquely human an emotion is perceived to be, the less it is ascribed to outgroup members [48,60]. This effect is thought to be independent of emotion valence. If collective responsibility for harm induces infrahumanization of the outgroup, then there should be a stronger negative relationship between emotion humanness and outgroup typicality in the harm responsible condition than in the harm not responsible condition.

However, we suggest an alternative hypothesis based on our findings of Studies 1 and 2. We suggest that victims of harm are perceived as experiencing negative emotions to a greater extent and positive emotions to a lesser extent regardless of emotion humanness or whether or not one feels collective responsibility for the harm. This trend is broadly in line with some theories of empathy, which suggest that we might over-attribute negative emotions to those we see as experiencing harm as a means of moral compensation or a social desirability towards signaling empathy [63,64]. Thus, in Study 3, we expected a stronger negative relationship to emerge between emotion valence and outgroup emotion typicality in both the harm responsible and harm not responsible conditions than in the no harm condition.

## Method

### Participants

Our primary analysis was done using emotion items rather than participants. A sensitivity analysis using G*Power indicated that including 64 emotions in the regression models would allow us to detect a medium effect size (*f² = *.15), with an alpha of .05 and a power of .85. Online recruitment for this study occurred between the 15^th^ and the 23^rd^ of February 2023. We recruited 252 participants (84 per condition) to ensure the average scores for emotion valence, humanness, and outgroup typicality were reliable. All participants were adult UK nationals currently residing in the UK who were fluent in English and had an approval rating of at least 95% on Prolific. Among participants, 132 identified as female, 117 as male, two as nonbinary, and one preferred not to indicate their gender identity. Ages ranged from 18 to 81 (*M = *43.9, *SD* = 15.2). As in the previous studies, an effort was made to ensure the sample was balanced in political orientation, with 127 participants being left-leaning and 125 being right-leaning. Differences in political orientation did not influence the key effects of interest, with separate analyses for left and right leaning participants yielding the same results as those reported. Data submitted by five participants were omitted and replaced due to missing responses.

### Design and analysis strategy

All participants responded to the same emotion humanness, emotion valence, and outgroup emotion typicality measures. Participants were randomly allocated to one of three between-subject conditions: harm responsible, harm not responsible, and no harm. This study, therefore, took a 3 (condition: harm responsible, harm not responsible, and no harm; between) X emotion humanness (continuous; within) X emotion valence (continuous; within) mixed design, with perceived emotional experiences of residents of Lagos as the dependent variable.

Previous work on infrahumanization following harm suggests a stronger negative relationship between emotion humanness and outgroup emotion typicality ratings should emerge in the harm responsible condition compared to the harm not responsible and no harm conditions, irrespective of emotion valence. However, we suggest that a stronger negative relationship between emotion valence and outgroup emotion typicality ratings should emerge in the two harm conditions compared to the no harm condition, irrespective of emotion humanness.

We followed the analysis plan Castano and Giner-Sorolla [48] used to test these two hypotheses. We first created average scores for emotion humanness, emotion valence and outgroup emotion typicality for each condition separately. This data transformation process can be seen in the main analyses Rscript included in the online supplementary materials. These average scores for individual emotions were then treated as observations in the main analyses, where we regressed average emotion humanness and valence scores onto outgroup emotion typicality ratings. In testing for infrahumanization following harm, our three models were:

1. Outgroup typicality (DV) ~ emotion humanness (IV) in the harm responsible condition
2. Outgroup typicality (DV) ~ emotion humanness (IV) in the harm not responsible condition
3. Outgroup typicality (DV) ~ emotion humanness (IV) in the no harm condition

The coefficients of each model were then compared with Fisher’s Z transformation using the *cocor* package in *RStudio* [65–67]. This process determined whether a significantly stronger negative relationship existed between emotion humanness and outgroup emotion typicality ratings for the harm responsible condition than the harm not responsible condition.

The above steps were repeated to test our alternative prediction, with emotion valence entered as the predictor variable rather than emotion humanness. Thus, the three models compared when testing our alternative prediction of negative affect driving emotion perception in harmed outgroup members were as follows:

1. Outgroup typicality (DV) ~ emotion valence (IV) in the harm responsible condition
2. Outgroup typicality (DV) ~ emotion valence (IV) in the harm not responsible condition
3. Outgroup typicality (DV) ~ emotion valence (IV) in the no harm condition

We expected a stronger negative relationship between emotion valence and outgroup typicality ratings in the two harm conditions compared to the no harm condition, regardless of emotion humanness.

To test for the effects of humanness independently of valence (models 1–3) and valence independently of humanness (models 4–6), we needed to ensure the two constructs were orthogonal in the data. Otherwise, we would need to control for the effects of one as a covariate when measuring the other and vice versa. Before conducting the regression analyses, we checked for correlations between ratings of emotion humanness and emotion valence. No significant correlations were found between emotion humanness and emotion valence among data submitted by participants in the harm responsible condition (*r* = -.03, *p* = .814, BF_01_ = 6.24), the harm not responsible condition (*r* = -.06, *p* = .628, BF_01_ = 5.74), or in the no harm condition (*r* = -.03, *p* = .817, BF_01_ = 6.24). These nonsignificant correlations imply that emotion humanness and emotion valence were orthogonal in our dataset. Thus, partial regressions were unnecessary [68]. Therefore, a single predictor and outcome variable were entered into each regression model.

As in Studies 1 and 2, we also conducted one-way ANOVAs to check the data from the manipulation checks to be sure that the perceived extent of harm and sense of collective responsibility for the harm were manipulated as intended between the conditions.

### Materials

**Emotion Humanness.** After briefly describing how some emotions might be considered unique to humans and some we share with other animals, participants were given the following prompt: *“To what extent do you think each emotion is experienced by humans and other animals equally, or by humans only?”.* Participants then rated each of the 64 emotions along the dimension of humanness using an unmarked sliding scale ranging from 0 (*Humans and other animals equally*) to 100 (*Humans only*), with the sliders initially fixed at the scale’s midpoint. An attention check (*Please indicate ‘Humans only’)* was included roughly halfway through the scale.

**Emotion Valence.** After briefly describing how some emotions might be considered positive to experience and others negative to experience, participants were given the following instruction: *“How does each emotion make people feel?”.* Participants then rated each of the 64 emotions on how positively or negatively valenced they were using an unmarked sliding scale ranging from 0 (*Extremely negative*) to 100 (*Extremely positive*). The sliders were initially fixed at the scale’s midpoint (*Neither positive nor negative*) for each item. An attention check (*Please indicate ‘Extremely negative’*) was included approximately halfway through the scale.

**Filler Task.** A filler task was included before the experimental stimuli to minimize carryover effects from the initial emotion-scoring tasks. An example of a *divergent uses task* [69,70], the filler task involved asking participants to think of as many uses for a brick as possible in one minute, entering their responses into an open text box.

**Vignettes.** We used the same climate change vignettes for each condition described in Study 1, which can be seen entirely in the online supplementary materials.

**Outgroup Emotion Typicality Ratings.** After reading the vignettes, all participants were asked to respond to 64 outgroup emotion typicality items. Each item read as “*Residents of Lagos typically feel X*”, with X being one of the 64 emotions. Participants responded to all 64 emotion typicality items using an unmarked sliding scale ranging from 0 (*Not at all*) to 100 (*Very much so*), with the sliders initially fixed at the midpoint of the scale (*Somewhat*). The 64 emotion items were presented randomly, apart from an attention check (*Residents typically: please indicate ‘not at all*’), which appeared approximately halfway through the task.

An effort was made to include a selection of emotions balanced across perceived humanness and valence based on pretest data collected by Enock et al. [39]. On average, emotions considered unique to humans were significantly higher in humanness than those rated as shared with other animals, but these categories did not differ in how positively or negatively valenced they were. Likewise, the average valence of emotions rated as positive to experience was significantly more positive than those rated as negative to experience, but positive and negative emotions did not differ in how uniquely human they were perceived to be. The emotions used in Study 3 were *admiration*, *agitation*, *amusement*, *anger*, *astonishment*, *attachment*, *attraction*, *awe*, *bitterness*, *compassion*, *confusion*, *contentment*, *depression*, *desire*, *disappointment*, *disenchantment*, *disgust*, *disillusion*, *dread*, *empathy*, *enjoyment*, *envy*, *euphoria*, *excitement*, *fascination*, *fear*, *fury*, *gratitude*, *grief*, *guilt*, *happiness*, *hate*, *hope*, *horror*, *humility*, *irritation*, *jealousy*, *joy*, *loneliness*, *love*, *lust*, *optimism*, *pain*, *panic*, *passion*, *pleasure*, *pride*, *relief*, *remorse*, *repulsion*, *resentment*, *resignation*, *sadness*, *self-satisfaction*, *serenity*, *shame*, *shyness*, *sorrow*, *spite*, *surprise*, *sympathy*, *tenderness*, *terror,* and *triumph*.

**Manipulation Checks.** The same manipulation checks as those in Study 1 were used in the present study. These were included to ensure that the perceived extent of harm experienced by residents of Lagos and the sense of collective responsibility felt by participants were manipulated as intended.

### Procedure

Participants were informed that the study was designed to help understand how people ascribe emotions to different groups of individuals. After reading the information sheet, participants were asked to indicate their consent to participation. Brief demographic questions followed, including gender identity, age, political orientation, and checks for inclusion criteria.

All participants then completed the emotion humanness and emotion valence ratings for each of the 64 emotions. Half of the sample completed the humanness ratings first, and half completed the valence ratings first. In both the humanness and the valence rating tasks, participants responded to a total of 65 items. These were the 64 randomized emotion items and one attention check item, which always appeared approximately halfway through. The 65 items were displayed in blocks of 17 items at a time, with the final block containing 14 items for both the humanness and valence rating tasks. Participants then completed the filler task, in an effort to take their minds off the emotion rating tasks to minimize carryover effects. Participants then moved on to the experimental stimuli for the study, the vignettes. The procedure for the section of this study involving vignettes was identical to that of Study 1.

After the vignettes, all participants were asked to respond to the outgroup emotion typicality block. After completing the outgroup emotion typicality ratings, participants could provide optional feedback and were debriefed before finishing the study. Participation took an average of 19 minutes.

## Results

### Manipulation checks

**The Perceived Extent of Harm.** A one-way between-subjects ANOVA revealed a significant difference in the perceived extent of harm experienced by residents of Lagos as a result of climate change, *F*(2, 249) = 36.772, *p* < .001, *η*² = .23, BF_01_ = 6.24. Post hoc comparisons indicated no significant difference between the harm responsible (*M* = 89.3, *SE = *1.34) and harm not responsible conditions (*M* = 92.3, *SE = *1.36), *p* = .744, 95% CI [-9.14, 3.21], BF_01_ = 1.98. However, the perceived extent of harm was significantly lower in the no harm condition (*M* = 73.0, *SE *=* *2.48) than in either the harm responsible condition, *p* < .001, 95% CI [-23.53, -11.19], BF_01_ < 0.01, or the harm not responsible condition, *p* < .001, 95% CI [-26.49, -14.15], BF_01_ < 0.01. The perceived extent of harm was manipulated as intended between conditions in Study 3, as illustrated in the right panel of Fig 1.

**Sense of Collective Responsibility.** A one-way between-subjects ANOVA revealed a significant difference between conditions in the extent to which participants felt a sense of collective responsibility for the harm caused by climate change, *F*(2, 249) = 9.682, *p* = < .001, *η*² = .07, BF_01_ = 0.01. Post hoc comparisons revealed that participants in the harm responsible condition felt significantly more collective responsibility for the harm (*M* = 60.2, *SE* = 2.46) than did those in the harm not responsible condition (*M* = 49.2, *SE* = 2.84), *p* = .009, 95% CI [2.14, 19.86], BF_01_ = .12 and those in the no harm condition (*M = *44.4, *SE* = 2.48), *p* < .001, 95% CI [6.91, 24.64], BF_01_ < 0.01. There was no significant difference in collective responsibility between the harm not responsible and no harm conditions, *p* = .586, 95% CI [-4.09, 13.64], BF_01_ = 2.86. Thus, participants’ sense of collective responsibility was manipulated as intended between conditions in Study 3, as seen in the right panel of Fig 2.

### Emotion ratings

Before conducting the main analyses, we checked whether emotion humanness and valence correlated. There were no significant correlations between ratings of emotion humanness and emotion valence in the harm responsible condition, *r* = -.030, *p* = .814, BF_01_ = 6.24; the harm not responsible condition, *r* = -.062, *p* = .628, BF_01_ = 5.71; or the no harm condition, *r* = -.030, *p* = .817, BF_01_ = 6.24. Thus, we did not need to control for potential effects of valence when examining the relationships between outgroup typicality and emotion humanness, and it was not necessary to control for potential effects of humanness when examining the relationships between outgroup typicality and emotion valence.

**Testing for Infrahumanization Following Harm.** A series of three linear regressions were conducted in which the relationship between emotion humanness and outgroup emotion typicality ratings was examined. The first model measured the relationship in the harm responsible condition, for which *Bitterness* was identified as highly influential using Cook’s distance and, thus, omitted (leaving a final *N* = 63). A significant negative relationship for the harm responsible condition was found, *F*(1, 61) = 5.667, *b *= -.128, *p* = .02, *R*^*2*^* = *.09, BF_01_ = 0.38. The second model measured the relationship in the harm not responsible condition, with *Bitterness*, *Disenchantment* and *Disillusion* identified as being highly influential and, therefore, omitted (leaving a final *N* = 61). A significant negative relationship was also found for the harm not responsible condition, *F*(1, 59) = 7.84, *b *= -.202, *p* = .007, *R*^*2*^* = *.12, BF_01_ = 0.16.

The third model measured the relationship in the no harm condition, for which *Optimism* and *Terror* were excluded because they were identified as highly influential (leaving a final N = 62). A significant negative relationship between emotion humanness and outgroup emotion typicality ratings was also found in the no harm model, *F*(1, 60) = 5.168, *b* = -.150, *p* = .027, *R*^*2*^* = *.08, BF_01_ = 0.46. In all three of the conditions, the more human an emotion was, the less residents of Lagos were seen as typically experiencing it.

**Fig 5:**
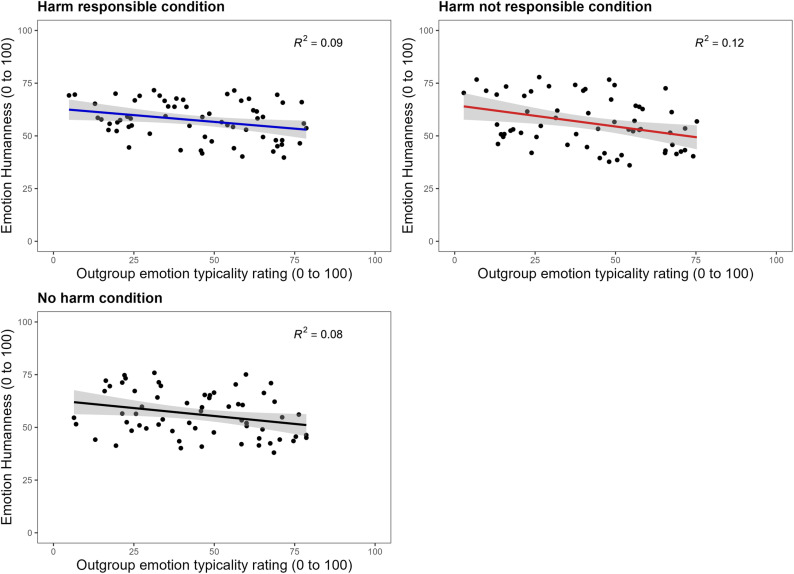
Results of Study 3: Testing for infrahumanization following harm. The three regression models with emotion humanness as the predictor and emotion typicality for outgroup members (residents of Lagos) as the outcome are displayed. The small negative relationship between these variables did not differ between the three conditions. *Note:* Grey shading around the slope represents 95% CI.

Our main question of interest was whether the regression coefficients differed significantly between conditions. Fisher’s *Z* transformations were performed using the *cocor* independent groups function in *RStudio*. These tests revealed no significant difference in the slopes between the harm responsible model (*b* = -.128, *N* = 63) and harm not responsible model (*b* = -.202, *N* = 61), *z = *0.413, *p* = .68, *Cohen’s q = *.08. Additionally, no difference in the relationship was found between the no harm model (*b* = -.150, *N* = 62) and the harm responsible model (*z* = 0.122, *p* = .90, *Cohen’s q = *.02), or between the no harm and harm not responsible models (*z* = 0.291, *p* = .77, *Cohen’s q = *.05). The extent to which perceptions of emotion humanness predicted outgroup emotion typicality ratings did not significantly differ between the three harm conditions. We did not replicate prior work, which suggests that outgroup members are seen as experiencing uniquely human emotions to a lesser extent when a sense of collective responsibility for harming them is elicited, compared to when it is not. The three models with emotion humanness as the predictor and outgroup emotion typicality as the outcome are plotted in Fig 5.

**Testing for Negative Affect when Harmed.** We ran another series of three linear regressions to test our alternative explanation whereby outgroup members are seen as experiencing negative emotions to a greater extent when they are perceived as being harmed than when they are not. In each model, emotion valence was entered as the predictor and outgroup emotion typicality ratings as the outcome. The first regression model measured the relationship in the harm responsible condition, for which the emotions *Guilt*, *Shame*, and *Love* were identified as highly influential to the model and omitted (leaving a final *N* = 61).

A significant negative relationship was found in the harm responsible model, *F*(1, 59) = 52.51, *b* = -.198, *p* < .001, *R*^*2*^* = *.47, BF_01_ < 0.01. The second model measured the relationship in the harm not responsible condition, with *Guilt*, *Love,* and *Shame* again identified as a highly influential and omitted (leaving a final *N* = 61). A significant negative relationship was also found for the harm not responsible model, *F*(1, 59) = 116.3, *b* = -.314, *p* = < .001, *R*^*2*^* = *.66, BF_01_ < 0.01. In the third model, the no harm model, the emotions *Euphoria,* and *Grief* were identified as highly influential and omitted (leaving a final *N* = 62). Unlike the two harm models, a significant positive relationship between emotion valence and outgroup emotion typicality ratings was found in the no harm model, *F*(1, 60) = 283.9, *b* = .329, *p* < .001, *R*^*2*^* = *.83, BF_01_ < 0.01.

**Fig 6:**
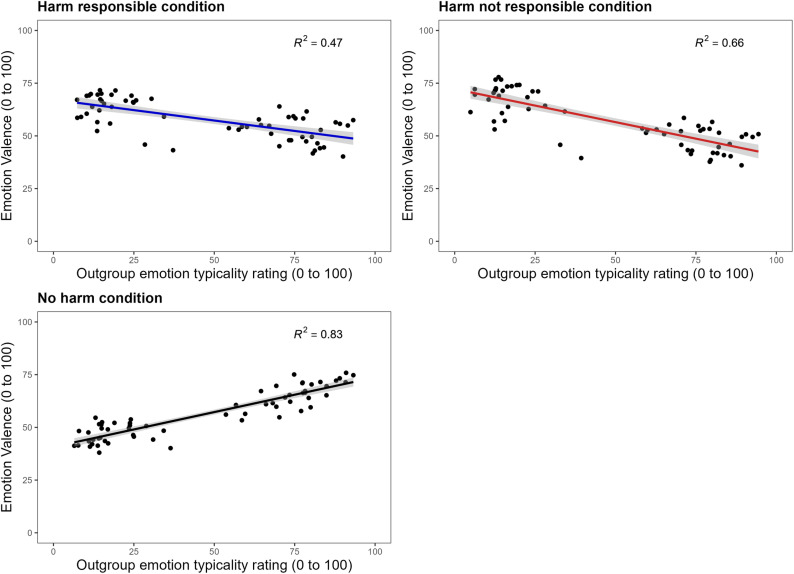
Results of Study 3: Testing for negative affect when harmed. The three regression models with emotion valence as the predictor and emotion typicality for outgroup members (residents of Lagos) as the outcome are displayed. As predicted, a negative relationship emerged in both the harm responsible and harm not responsible conditions, with each found to differ significantly from the positive relationship that emerged in the no harm condition. *Note:* grey shading around the slope represents 95% CI.

A Fisher’s Z transformation was performed using the *cocor* independent groups function in *RStudio* to test whether the relationship between emotion valence and the perceived typicality of emotions in residents of Lagos significantly differed between the two harm conditions and the no harm condition. The difference between the negative relationship in the harm responsible model (*b *= -.198, *N* = 61) and the positive relationship in the no harm model (*b* = .329, *N* = 61) was significant, *z* = 2.934, *p* = .003, *Cohen’s q = *.54. A significant difference was also found between the negative relationship in the harm not responsible model (*r* = -.314, *N* = 61) and the positive relationship in the no harm model, *z* = 3.609, *p* < .001, *Cohen’s q = *.67. As predicted, participants rated outgroup members as typically experiencing negative emotions to a greater extent, and positive emotions to a lesser extent, when harm to them is made salient. No significant difference was found between the harm responsible and harm not responsible models, *z* = 0.672, *p* = .502, *Cohen’s q = *.13. The three regression models with emotion valence as the predictor and outgroup emotion typicality as the outcome are plotted in Fig 6.

When testing both hypotheses, the same results as those reported above were found when all 64 emotions were included in the regression models, when any emotion identified as highly influential in one model was removed from all three models (*N* = 59 in all models), and when performing Fisher’s Z transformations on respective Pearson correlation coefficients rather than regression coefficients. Results for these alternative analyses are reported in the online supplementary materials.

## Study 3 Discussion

Despite having a more sensitive design than in Studies 1 and 2, we again found no evidence that feeling collective responsibility for harm induces infrahumanization of the outgroup. Again, we found evidence for an alternative pattern of emotion attribution that aligned with the results of Studies 1 and 2. As predicted, we found that outgroup members were seen as experiencing negative emotions to a greater extent and positive emotions to a lesser extent when harm against them was made salient.

## General discussion

Previous research has suggested that subtle dehumanization can occur as an outcome of, or response to, intergroup harm. Castano and Giner-Sorolla [48] and Čehajić et al. [50] each reported that participants are more likely to deny members of a group uniquely human emotions when they believe their ingroup to be responsible for harming them. Across three well-powered and pre-registered studies, we sought to measure whether these influential findings replicate in two new intergroup contexts: the harm caused by climate change and the harm caused by fast fashion. In all three studies, we manipulated the extent to which UK residents felt responsible for the harm caused to another group. In Studies 1 and 2, we presented participants with 16 emotions that varied in how human they are perceived to be and how positive they are to experience. We found no evidence for the hypothesis that feeling responsible for harm reduces the extent to which participants attribute uniquely human emotions to outgroup members. The robustness of these null effects was further supported by Bayesian analyses, which provided evidence exceeding moderate support for the null hypothesis when comparing the harm responsible and harm not responsible groups in their attribution of negative uniquely human emotions (BF₀₁ = 5.10 to 5.74). For positive uniquely human emotions, Bayes factors approached moderate support for the null (BF₀₁ = 2.53 to 2.67). Instead, we found evidence for a much simpler pattern of emotion attribution. When the harm experienced by a group was made salient, participants believed members of that group typically experience more negative and less positive emotions, regardless of emotion humanness and their sense of collective responsibility.

One plausible reason for failing to replicate previous findings is that we used an insufficiently sensitive design. In Studies 1 and 2, we broadly followed the methods of Čehajić et al. [50]. Like almost all studies on infrahumanization, Čehajić and colleagues presented participants with a relatively small number of emotions (16) that varied categorically in perceived humanness and valence. While this approach is prevalent in the empirical literature [5,11,27,28,39,71–75], it has recently been criticized as potentially obscuring evidence for subtle dehumanization such as infrahumanization effects [42,60].

In Study 3, we utilized a design incorporating many emotions in a manner more similar to that used by Castano and Giner-Sorolla [48]. In this study, we asked participants to rate 64 emotions along the dimensions of humanness, valence, and typicality of the outgroup. We then compared the strength of the relationship between these variables between conditions. The same participants who responded to the dependent variable provided the humanness and valence scores, with mean scores reflecting their subjective categorization of each emotion. As Castano and Giner-Sorolla [48] noted, using continuous variables also allows for more emotions to be included in the measures (p.806), providing more reliable mean scores for each emotion variable. We found no evidence that participants were more likely to infrahumanize the outgroup in the harm responsible condition. Instead, converging with the results of studies 1 and 2, we found that participants perceive outgroup members to experience negative emotions more strongly when harm they have experienced is made salient. While the design of Study 3 was well-powered and robust, it remains possible that methodological differences between Castano & Giner-Sorolla’s work may explain why we could not conceptually replicate their findings. Future work could help clarify whether apparent infrahumanization following harm may emerge only with specific methods, if at all.

It is interesting to consider why we did not observe the same emotion attribution trends reported by Castano and Giner-Sorolla [48] and Čehajić et al. [50]. One possibility is that infrahumanization following harm may occur in some intergroup contexts but not others. Castano and Giner-Sorolla [48] tested their hypothesis with the harm committed by humans against hypothetical aliens and the real-world harm inflicted on Indigenous Americans and Aboriginal Australians by European colonizers. Čehajić et al. [50] tested their hypothesis in the contexts of the Serbian genocide against Bosnian Muslims in the 1990s and the colonial harm inflicted by Europeans on Indigenous Chileans in colonial times. On the other hand, we tested our hypotheses with the more contemporary contexts of harm caused to people by climate change and exploitation in fast fashion supply chains. While departing from the colonial and genocidal intergroup contexts of previous work, the contexts we examine are often mentioned in the media and may feel more current, tangible, and relatable to people. Indeed, these are cases of ongoing suffering and exploitation that many individuals actively and frequently contribute to today.

Despite our manipulation checks having broadly demonstrated that the perceived harm and sense of collective responsibility for harm were manipulated between participant conditions as intended, further contextual differences might explain why the previously reported effects failed to replicate. It would be interesting for future research to investigate these possible differences (e.g., the impact of past harm compared to ongoing harm on intergroup perceptions) and in which contexts infrahumanization following harm, or perceiving negative affect following harm, is more likely to occur. The scale of harm could be something for future research to examine in tandem, to help understand whether apparent infrahumanization may occur in response to certain harms but not others. As we only tested for infrahumanization, examining other forms of dehumanization in post-harm intergroup contexts would also be an interesting avenue for future research. For example, it remains possible that while infrahumanization might not occur, explicit or linguistic dehumanization or a denial of human rights and equal status, as described by Luft [44,45] and Smith [16,47,76], could emerge in post-harm contexts. Relatedly, it would be interesting for future research to examine how behaviors associated with empathy more generally might help to explain the trends observed in our studies. For example, our participants could have over-attributed distress related emotions to the harmed residents of Lagos or fast fashion workers in our vignettes as a means of moral compensation or social desirability towards signaling empathy [63,64].

Along with examining other interpretations of post-harm dehumanization in the literature, recruiting a more representative sample to check how demographic characteristics might also affect these intergroup perceptions could be interesting for future research. Participants in our studies were from a Western, Euro-centric society (the UK), where social desirability towards explicitly anti-prejudicial norms may have influenced how they responded to our intergroup measures. This tends to be common weakness of dehumanization research more generally, which our work inherits as we aimed to conceptually replicate previous trends and methods. However, it is worth noting that measures of infrahumanization are assumed to be subtle in that participants are likely unaware that they are being asked to indicate how human they perceive others to be. While not the aim of the current research, examining intergroup perceptions in a wider range of intercultural contexts where the line between ingroup and outgroup may vary in saliency, power differences, and historical factors, could help in clarifying the extent to which findings of dehumanization research are generalizable. Future research could also aim to account for the possible moderating role of political orientation on infrahumanization [12,15]. While we checked that our results did not differ from those reported when controlling for this variable our analyses were not sufficiently powered to be conclusive.

It is worth pointing out that while the results of Castano and Giner-Sorolla [48] and Čehajić et al. [50] are regularly cited as evidence for the claim that infrahumanization follows harm, some of the results they report are not especially strong. For example, the crucial interaction between the humanness of the emotion terms and conditions reported by Castano and Giner-Sorolla [48] is statistically significant in their Study 2 but only marginally significant in Study 1, reported as “*p <.06*” (p. 808). In Čehajić et al. [50], the results are considerably weaker. The crucial interaction between group membership and emotion attribution is marginally significant in their Study 1 (*p = *.08) and nonsignificant in Study 2 (*p = *.83). Against what is widely considered statistically appropriate, the authors break down these nonsignificant interactions into simple comparisons and report the pattern of results that they predicted. Thus, there may be many intergroup contexts in which these small effects are difficult, or even impossible, to detect.

A trend observed In Study 2 of Castano and Giner-Sorolla [48] that we also found in our Study 3 is that in all conditions, the more uniquely human an emotion is considered to be, the less outgroup members were seen as typically experiencing it. The reason for this trend remains unclear, and any suggestion as to why it occurred beyond the specific constructs examined remains speculative. One possibility is that this result suggests the overall infrahumanization of the outgroup. However, as neither of these studies included a measure of ingroup emotion attribution, we cannot rule out the possibility that the pattern of emotion attribution would be similar for ingroups and outgroups. It would be valuable to explore these questions further in future research. We found replication issues when using the same selection of emotions as previous work claiming to find evidence of infrahumanization following harm, raising concerns around the internal validity of the stimuli selection and associated constructs that are supposedly being measured. Moreover, it has been shown that the stimuli used in wider dehumanization research is often confounded by the influences of social desirability and intergroup preference [37–40,42]. Therefore, it is important that future research exercises caution when examining infrahumanization, or other subtle forms of apparent dehumanization.

Taken together, our findings suggest that the tendency to infrahumanize groups when we are responsible for harm against them is less common than often assumed. Importantly, we are not questioning whether dehumanization can sometimes occur more broadly as a response to harm. Many theorists, including Smith [16,47] and Luft [44,45], have suggested that dehumanization may occur as a means by which to justify harm. Broadly in line with this view, perpetrators of harm sometimes refer to their victims as less than human in qualitative interviews [46]. Instead, we suggest that the extent to which dehumanization following harm reveals itself as a denial of uniquely human emotions may be less widespread than often assumed. Given the real-world importance of understanding the motivations and psychological processes involved in intergroup hostility and harm, social psychology must rely on valid and replicable findings going forward.

## Supporting information

S1 File Supplementary materials - post-harm infrahumanization. Supplementary materials includes the vignettes used in all studies, equivalence tests, main effects and two-way interactions for Studies 1 & 2, and alternative analyses for Study 3.
(DOCX)
