# Supplementary material for: Does feeling collective responsibility for intergroup harm lead to infrahumanization?
Source: PLoS One. 2025 May 29;20(5):e0323292. doi: 10.1371/journal.pone.0323292 (PMC12121804; doi:10.1371/journal.pone.0323292)
Supplement: S1 File — Supplementary materials includes the vignettes used in all studies, equivalence tests, main effects and two-way interactions for Studies 1 & 2, and alternative analyses for Study 3. (DOCX) [file pone.0323292.s001.docx]

**Supplementary materials for:**

Does feeling collective responsibility for intergroup harm lead to infrahumanization?

**Authors:** Robert A. Brennan^1, 2^*, Florence E. Enock^1, 3^, Carl J. Bunce^1, 4^ & Harriet Over^1^

^1^ Department of Psychology, University of York, York YO10 5DD, United Kingdom

^2^ The Institute of Future Media, Democracy and Society, Dublin City University, Dublin D09 Y074, Ireland

^3^ The Alan Turing Institute, British Library, 96 Euston Road, London NW1 2DB, United Kingdom

^4^ School of Psychology and Clinical Language Sciences, University of Reading, Reading, RG6 6ET, United Kingdom

* Correspondence to Robert A. Brennan: [robert.a.brennan@dcu.ie](mailto:robert.a.brennan@dcu.ie) ; [brennan.rob.a@gmail.com](mailto:brennan.rob.a@gmail.com)

**List of contents**

[Method: Vignettes in Full 4](#_Toc192772707)

[Studies 1 and 3: Climate Change Context 4](#_Toc192772708)

[Study 2: Fast Fashion Context 9](#_Toc192772709)

[Results: Main Effects and Two-Way Interactions 14](#_Toc192772710)

[Study 1: Testing for Infrahumanization of Outgroup Members Harmed by Climate Change 14](#_Toc192772711)

[Emotion Ratings. 14](#_Toc192772712)

[Study 2: Testing for Infrahumanization of Outgroup Members Harmed by Fast Fashion 15](#_Toc192772713)

[Emotion Ratings. 15](#_Toc192772714)

[Comparing Alternative Analyses for Study 3 18](#_Toc192772715)

[Alternative Analyses When Testing for Infrahumanization Following Harm 18](#_Toc192772716)

[Alternative Analyses when Testing for Negative Affect when Harmed. 20](#_Toc192772717)

[Equivalence testing for Studies 1 and 2 22](#_Toc192772718)

**List of tables**

[**Table S1.** Initial climate change harm vignettes for the harm responsible and harm not responsible conditions 5](#_Toc192773149)

[**Table S2.** Harm responsible vignettes in the climate change context 6](#_Toc192773150)

[**Table S3**. Harm not responsible vignettes in the climate change context 7](#_Toc192773151)

[**Table** **S4**. No harm vignettes in the climate change context 8](#_Toc192773152)

[**Table** **S5**. Initial fast fashion harm vignettes for the harm responsible and harm not responsible conditions 10](#_Toc192773153)

[**Table** **S6**. Harm responsible vignettes in the fast fashion context 11](#_Toc192773154)

[**Table** **S7**. Harm not responsible vignettes in the fast fashion context 12](#_Toc192773155)

[**Table** **S8**. No harm vignettes in the fast fashion context 13](#_Toc192773156)

[**Table** **S9**. Results of regression models when testing for infrahumanization following harm using different analyses 18](#_Toc192773157)

[**Table** **S10**. Comparing the regression coefficients between the different conditions when testing for infrahumanization following harm using different analyses 19](#_Toc192773158)

[**Table** **S11**. Results of regression models using different analyses when testing for negative affect when harmed 20](#_Toc192773159)

[**Table** **S12**. Comparing the regression coefficients between the different conditions when testing for negative affect when harmed, using different analyses 21](#_Toc192773160)

[**Table** **S13**. Equivalence testing results for Studies 1 and 2. 23](#_Toc192773161)

# Method: Vignettes in Full

## Studies 1 and 3: Climate Change Context

Initial vignettes for both the **harm responsible** and **harm not responsible** conditions detailing the harm climate change has on residents of Lagos are displayed in Table S1.

Participants in the **harm responsible** condition then read the paragraphs in Table S2, detailing the contributions of UK residents to climate change. Participants in the **harm not responsible** condition instead read the paragraphs in Table S3, emphasizing the contribution of multinational Big Oil companies.

Participants in the **no harm** condition read only four paragraphs in total, which are displayed in Table S4. These contained no mention of climate change or harm and instead detailed aspects of the local culture, economic activities, and lifestyle of residents of Lagos.

All paragraphs were displayed one at a time, and an 8 to 10-second delay was applied before participants could move on to the next.

Table S1. Initial climate change harm vignettes for the harm responsible and harm not responsible conditions

| Paragraph 1: | Although climate change is expected to affect all countries, some regions are particularly vulnerable. Lagos in Nigeria is one such region that has already been impacted. The largest city in Nigeria, as well as the most populous on the African continent, the residents of Lagos are considered to be at extreme risk from the harmful effects of climate change. |
| --- | --- |
| Paragraph 2: | Lagos currently hosts a thriving economy and reached a Gross Domestic Product (GDP) of USD 136 billion in 2017, about a third of the GDP of the whole of Nigeria. The city is a major transportation hub, with multiple ports and a major international airport. Lagos is also a regional hub for the high-tech industry. |
| Paragraph 3: | As climate change progresses, residents of Lagos have experienced increasingly more hot days and droughts, as well as higher humidity. Rising temperatures have enhanced the capacity of Anopheles mosquitoes to spread malaria and it is thought that the prevalence of other diseases has also increased. Ailments like measles, meningitis, heat rashes, dehydration and respiratory problems have all been linked to severe heat and high relative humidity. |
| Paragraph 4: | Climate change has already impacted the city's economy. Lagos is especially vulnerable because it is located on the Gulf of Guinea. Rising sea levels are causing coastal erosion and contaminating drinking water sources. This further harms local agriculture and has damaged the country's important fishing industry. |
| Paragraph 5: | The resulting job losses have caused lower levels of income and a poorer standard of living for those whose livelihoods depend on such industries. Experts fear the extent of the damage that climate change has on Lagos, with displacement and loss of life already being experienced by some of its residents. |

Table S2. Harm responsible vignettes in the climate change context

| **Paragraph 1:** | The UK is historically one of the world's greatest contributors to global warming. This is because of our long history of generating greenhouse gas emissions which began during the Industrial Revolution in the eighteenth and nineteenth centuries. |
| --- | --- |
| **Paragraph 2:** | Richer countries like the UK are responsible for far more carbon pollution than the world's poorer countries. Today, the average carbon footprint per person in the UK is at around 12.7 metric tonnes of carbon dioxide emissions per year, nearly three times more than the worldwide average. |
| **Paragraph 3:** | Considering the significant impact that residents of high-polluting countries such as the UK have, global average temperatures could increase by 4°C by the end of the century. Despite recent efforts to reduce carbon emissions in the UK, there is still much room for improvement and we are all collectively responsible. |
| **Paragraph 4:** | The average UK resident contributes a substantial amount of support for industries that are spearheading global warming. With high rates of meat and dairy consumption as well as the excessive prevalence of fast fashion and single-use products, the consumer habits of UK residents are typically unsustainable. Moreover, 61% of surface transport emissions in the UK are from private cars and UK residents also create high CO2 emissions from international flights. |

Table S3. Harm not responsible vignettes in the climate change context

| **Paragraph 1:** | A recent report found that, since 1988, just 100 international companies have been collectively responsible for 71% of global greenhouse gas emissions. Further, just 25 corporations and state-owned entities were responsible for more than half of global industrial emissions in that same period. |
| --- | --- |
| **Paragraph 2:** | The Carbon Disclosure Project has recently presented data that "pinpoints how a relatively small set of fossil fuel producers may hold the key to systemic change on carbon emissions". Considering the significant impact that high polluting companies have, global average temperatures could increase by 4°C by the end of the century. |
| **Paragraph 3:** | Despite recent calls to reduce carbon emissions globally, there is still much room for improvement and companies with the highest global greenhouse gas emissions must take responsibility. Most of these are coal and oil-producing companies and include ExxonMobil, Shell, Chevron, Gazprom, and the Saudi Arabian Oil Company. Such companies emit large amounts of greenhouse gases at each stage of their supply chain. Locating fossil fuel reserves often involves offshore seismic shocks and seabed drilling. |
| **Paragraph 4:** | The processing and transportation of fossil fuels also generate substantial levels of emissions. The scale of historical emissions associated with these fossil fuel producers is large enough to have already contributed significantly to climate change, according to the report. |

Table S4. No harm vignettes in the climate change context

| **Paragraph 1:** | Many countries across the world have seen considerable economic development in recent decades. Lagos in Nigeria is one such region. The largest city in Nigeria, as well as the most densely populated on the African continent, the residents of Lagos are now thought to number over 10 million people. |
| --- | --- |
| **Paragraph 2:** | Lagos is often considered Africa's foremost urban centre and hub of regional, national, and global socio-economic and political activities. Lagos currently hosts a thriving economy and reached a Gross Domestic Product (GDP) of USD 136 billion in 2017, about a third of the GDP of the whole of Nigeria. The city is a major transportation hub, with multiple ports and a major international airport. Lagos is also a regional hub for high-tech industry. |
| **Paragraph 3:** | The residents of Lagos experience a tropical climate that tends to be hot all year round. The dry season is from November to March and the rainy season is from April to October. The hottest month tends to be March, with average temperatures around 30°C, and the coldest month tends to be August, with average temperatures around 26°C. Humidity in Lagos is generally high all year round, with an annual average relative humidity of 84.7%, ranging from 80% in March to 88% in June. |
| **Paragraph 4:** | Given that it is located on the Gulf of Guinea, local fishing and agricultural industries are central to the economy of the city and surrounding region, providing many jobs for local residents. The food and cultural scene also provide local jobs. Recreational activities take place along the Atlantic coastline of Lagos, where there are a number of beaches, including Elegushi Beach and Alpha Beach. |

## Study 2: Fast Fashion Context

Initial vignettes for both the **harm responsible** and **harm not responsible** conditions detailing the harm experienced by textile workers in fast fashion supply chains are displayed in Table S5.

Participants in the **harm responsible** condition moved on to read the paragraphs in Table S6, detailing the high rates of fast fashion consumption by UK residents, emphasizing how this is complacent in the harm experienced by textile workers, allowing it to continue. Participants in the **harm not responsible** condition instead read the paragraphs in Table S7, emphasizing how fast fashion companies' profit-driven CEOs and boards of directors are responsible for the harm experienced by textile workers in supply chains.

Participants in the **no harm** condition read only four paragraphs displayed in Table S8. These contained no mention of the exploitation or harm experienced by textile workers and instead described the history and prominence of fast fashion globally.

All paragraphs were displayed one at a time, and an 8 to 10-second delay was applied before participants could move on to the next.

Table S5. Initial fast fashion harm vignettes for the harm responsible and harm not responsible conditions

| **Paragraph 1:** | Fast fashion refers to the production of high volumes of clothing throughout the year. The fast fashion business model relies on a continuous demand for new clothes, offering cheap garments and ever-changing new ranges. The fast fashion industry allows extreme harm to be inflicted on the textile workers who work along the supply chains. |
| --- | --- |
| **Paragraph 2:** | So-called 'sweatshops' are particularly common in South and Southeast Asia. These factories manufacture products for the world's largest clothing brands. The workers in these factories are not typically offered legal protections or fair wages and basic human rights may be neglected. Moreover, gender-based violence, child labour and slavery have been documented within supply chains for fast fashion companies. |
| **Paragraph 3:** | Workers for fast fashion supply chains may live in dire socioeconomic conditions, struggling to afford necessities such as food and electricity, even when being expected to work an illegal number of hours. Physical and psychological injuries are widely documented, with no compensation schemes in place. It is estimated that 27 million people working in the fast fashion industry, from cotton farming to sweatshops, suffer work-related illnesses. |
| **Paragraph 4:** | Exposure to chemicals in farming and textile processing can cause skin and respiratory conditions. Hearing damage, repetitive strain injury, musculoskeletal injury, eye strain and lung disease from lint inhalation are all widespread amongst textile workers today. Furthermore, instances such as the collapse of Rana Plaza in Bangladesh which killed over 1,100 textile workers highlight the vulnerability of these workers who have no other choice but to work in unsafe, harmful conditions. |

Table S6. Harm responsible vignettes in the fast fashion context

| **Paragraph 1:** | One might wonder how fast fashion continues to dominate the UK fashion trade despite harmful practices being well-documented in its supply chain. As supplies aim to meet demand, it is the consumer that supports and maintains the fast fashion industry. The contribution of UK consumers is enormous. Despite exploitative practices being widely reported in the British media, most British people continue to buy fast fashion. |
| --- | --- |
| **Paragraph 2:** | UK consumers tend to buy clothes for the short term. It has been reported that a large number of people in the UK consider a garment worn only for a couple of months to be 'old'. The cycle of frequent purchasing, wearing, and disposing of clothes and other garments supports the high demand for the fast fashion model. This in turn enables the exploitation, injury, and deaths of textile workers to continue. |
| **Paragraph 3:** | A cross-party report by the House of Commons noted that UK residents buy more clothes per person than any other European country. These purchasing patterns perpetuate the continued exploitation of labourers in fast fashion supply chains. |

Table S7. Harm not responsible vignettes in the fast fashion context

| **Paragraph 1:** | One might wonder how fast fashion continues to dominate the global fashion trade despite such harmful practices within its supply chain. Like many global markets, it is the CEOs and directors of the companies involved that support and maintain the fast fashion industry. The contribution of each company is enormous, and the exploitation of their workers is often completely hidden from consumers. |
| --- | --- |
| **Paragraph 2:** | Boards of directors for fast fashion companies carefully market their products to normalise cheap, low-quality clothes. UK consumers are often unaware of the problems and have little option but to buy at least some of their clothes from these chains. The prioritization of profits by CEOs of multinational fast fashion companies allows for the exploitation, injury, and deaths of textile workers to continue. |
| **Paragraph 3:** | Multinational companies continue to push onwards, hiding workers' conditions from UK consumers, and increasing profits by each year. These practices perpetuate the continued exploitation of labourers in fast fashion supply chains. |

Table S8. No harm vignettes in the fast fashion context

| **Paragraph 1:** | Fast fashion refers to the production of high volumes of clothing throughout the year. The fast fashion business model relies on an endless demand for new clothes, offering inexpensive garments and ever-changing new ranges. Fast fashion brands are able to mass produce clothes at a low cost, meaning that consumers can update their wardrobes quickly and affordably. |
| --- | --- |
| **Paragraph 2:** | The accessibility of inexpensive new clothes is relatively new. Up until the about 1950s, a large amount of clothing was still made in the home, or in local factories and couture houses for families that could afford it. In the 1960s young people embraced cheaply made clothing to follow new trends. Fashion brands had to find ways to keep up with this increasing demand for affordable clothing, leading them to open textile mills across the world. |
| **Paragraph 3:** | Today, fast fashion is boosted even further with the rise of social media and celebrity culture. When a celebrity posts a photo wearing a new outfit, fast fashion brands rush to be the first to make a similar style accessible to the general public. |

# Results: Main Effects and Two-Way Interactions

## Study 1: Testing for Infrahumanization of Outgroup Members Harmed by Climate Change

### Emotion Ratings.

**Main Effects.** There was a significant main effect of emotion humanness, *F*(1, 249) = 307.608, *p* < .001, *η_p_*² = .55. Residents of Lagos were seen as typically experiencing emotions shared with other animals (*M =*57.4, *SE =*0.64) to a greater extent than uniquely human emotions (*M* = 48.6, *SE =*0.72), *p < .*001. There was also a significant main effect of emotion valence, *F*(1, 249) = 17.691, *p < .*001, *η_p_²* = .07, whereby participants rated residents of Lagos as experiencing positive emotions (*M* = 55.8, *SE* = 0.93) to a greater extent than negative emotions (*M* = 50.2, *SE* = 0.92), *p* < .001. There was no significant main effect of harm condition on emotion typicality ratings, *F*(2, 249) = 1.63, *p =*.198, *η_p_*² = .01.

**Condition*Humanness.** A significant two-way interaction between harm condition and emotion humanness was found *F*(2, 249) = 20.27, *p* < .001, *η_p_² =*.14. However, the pattern of results did not follow that predicted by infrahumanization theory. Residents of Lagos were not seen as experiencing uniquely human emotions any differently between the harm responsible condition (*M* = 48.7, *SE* = 1.24) and the harm not responsible condition (*M* = 47.8, *SE* = 1.24), *p* = 1. Uniquely human emotion ratings in the no harm condition (*M* = 49.4, *SE* = 1.24) did not differ from those in either of the harm conditions, both *p*s = 1. No difference in ratings of emotions shared with animals was found between the harm responsible condition (*M* = 60.0 *SE* = 1.11) and the harm not responsible condition (*M* = 58.3, *SE* = 1.11), *p* = .842. Compared to participants in the no harm condition (*M =*53.7, *SE =*1.11), emotions shared with other animals were rated significantly higher by those in both the harm responsible condition (*p < .*001) and the harm not responsible condition (*p* = .011).

**Condition*Valence.** A significant two-way interaction was found between harm condition and emotion valence, *F*(2, 246) = 57.714, *p* < .001, *η_p_*² = .32. No difference was found in ratings of positive emotions between participants in the harm responsible condition (*M =*53.14, *SE =*1.61) and those in the harm not responsible condition (*M =*49.80, *SE =*1.61), *p =*.433. Participants in the no harm condition (*M =*64.53, *SE =*1.61) rated positive human emotions higher than participants in either of the harm conditions, both *p*s < .001.

No difference in ratings of negative emotions was found between the harm responsible condition (*M =*55.6, *SE =*1.59) and the harm not responsible condition (*M =*56.3, *SE =*1.59), *p* = 1. Participants in both harm conditions rated negative emotions higher than those in the no harm condition (*M =*38.6, *SE =*1.59), both *p*s < .001.

**Humanness*Valence.** A significant two-way interaction between emotion humanness and emotion valence was found *F*(1, 249) = 275.124, *p* < .001, *η_p_² =*.53. No difference was found in how participants perceived residents of Lagos as experiencing positive emotions when they were uniquely human (*M* = 56.4, *SE* = 0.93) compared to when they were shared with other animals (*M* = 55.2, *SE* = 1.05), *p* = .074. In contrast, participants perceived residents of Lagos as experiencing negative emotions to a greater extent when they were shared with other animals (*M* = 59.5, *SE* = 1.07) than when they were uniquely human (*M* = 40.9, *SE* = 0.96), *p* < .001.

## Study 2: Testing for Infrahumanization of Outgroup Members Harmed by Fast Fashion

### Emotion Ratings.

**Main Effects.** A significant main effect of emotion humanness was found, *F*(1, 249) = 112.095, *p < .*001, *η_p_*² = .31. Emotions shared with other animals (*M =*47.0, *SE =*0.63) tended to be rated higher than uniquely human emotions (*M =*41.1, *SE =*0.77), *p* < .001. A significant main effect of emotion valence was also found, *F*(1, 249) = 348.403, *p* < .001, *η_p_*² = .58. Participants tended to rate textile workers as experiencing negative emotions (*M* = 58.0, *SE* = 0.90) to a greater extent than positive emotions (*M* = 30.2, *SE* = 1.06), *p* < .001. No main effect of harm condition was found, *F*(2, 249) = 0.63, *p* = .533, *η_p_*² = .01.

**Condition*Humanness.** A significant two-way interaction between harm condition and emotion humanness was found, *F*(2, 249) = 5.647, *p* = .004, *η_p_² =*.04. Contrary to the previous research on infrahumanization following harm, there was no significant difference in the extent to which participants attributed uniquely human emotions to textile workers between the harm responsible condition (*M =*41.4, *SE =*1.33) and the harm not responsible condition (*M =*39.2, *SE =*1.33), *p* = .74. Unlike in our Study 1 results, the extent to which participants attributed uniquely human emotions to textile workers did not differ between the no harm condition (*M* = 42.8, *SE* = 1.33) and either the harm responsible condition (*p* = 1) or the harm not responsible condition (*p* = .170). Similarly, the extent to which participants attributed emotions shared with other animals to textile workers did not differ between the harm responsible condition (*M =*48.0, *SE =*1.08) and the harm not responsible condition (*M =*46.9, *SE =*1.08), *p* = 1. Participants in the no harm condition (*M =*46.1, *SE =*1.08) did not rate shared emotions any differently than those in either the harm responsible condition (*p* = .627) or the harm not responsible condition (*p* = 1).

**Condition*Valence.** A significant two-way interaction was also found between harm and emotion valence, *F*(2, 246) = 29.730, *p* < .001, *η_p_*² = .19. No significant difference was found in the extent to which participants perceived textile workers as typically experiencing positive emotions between the harm responsible condition (*M* = 27.7, *SE* = 1.84) and the harm not responsible condition (*M* = 24.2, *SE* = 1.84), *p =*.524. Participants in no harm condition (*M* = 38.6, *SE* = 1.84) rated textile workers as typically experiencing positive emotions to a greater extent than those in either of the two harm conditions, both *p*s < .001. No significant difference was found in the extent to which participants believed textile workers typically experience negative emotions between the harm responsible condition (*M* = 61.7, *SE* = 1.55) and the harm not responsible condition (*M* = 61.9, *SE* = 1.55), *p =*1. Participants in each of the two harm conditions rated textile workers as experiencing negative emotions to a greater extent than those in the no harm condition (*M* = 50.3, *SE* = 1.55), both *p*s < .001.

**Humanness*Valence.** A significant two-way interaction between emotion humanness and emotion valence was found *F*(1, 249) = 563.328, *p* < .001, *η_p_² =*.68. Participants perceived textile workers in fast fashion supply chains as experiencing positive uniquely human emotions (*M* = 34.6, *SE* = 1.15) to a greater extent than positive emotions shared with other species (*M* = 25.7, *SE* = 1.10), *p* < .001. Participants also perceived textile workers as typically experiencing negative emotions shared with other animals (*M* = 68.3, *SE* = 1.02) to a greater extent than negative uniquely human emotions (*M* = 47.6, *SE* = 1.00), *p* < .001.

# Comparing Alternative Analyses for Study 3

## Alternative Analyses When Testing for Infrahumanization Following Harm

Alternative results when deciding which highly influential emotions were excluded (if any) are displayed in Table S9, and associated main results are displayed in Table S10.

Table S9. Results of regression models when testing for infrahumanization following harm using different analyses

| Analysis | Condition | *F* | *df* | *b* | *p* | *R^2^* | *r** |
| --- | --- | --- | --- | --- | --- | --- | --- |
| 64 emotions in all models | **HR** | 4.334 | 1,62 | -.112 | .042 | .07 | -.256 |
|  | **HNR** | 3.18 | 1,62 | -.126 | .08 | .05 | -.221 |
|  | **NH** | 2.581 | 1,62 | -.107 | .113 | .04 | -.2 |
| Model-by-model | **HR** | 5.667 | 1,61 | -.128 | .02 | .09 | -.292 |
|  | **HNR** | 7.84 | 1,59 | -.202 | .007 | .12 | -.343 |
|  | **NH** | 5.168 | 1,60 | -.15 | .027 | .08 | -.282 |
| 59 emotions in all models | **HR** | 7.231 | 1,57 | -.156 | .009 | .11 | -.336 |
|  | **HNR** | 5.636 | 1,57 | -.18 | .021 | .09 | -.3 |
|  | **NH** | 2.878 | 1,57 | -.123 | .095 | .05 | -.219 |

*Notes: HR =*Harm responsible condition; *HNR* = Harm not responsible condition; *NH* = No harm condition*; b* = coefficient of the predictor variable (emotion humanness); r** =*Pearson correlation coefficient between emotion humanness and outgroup emotion rating. No meaningful difference can be seen between the different analyses. Analysis with 64 emotions in all models included all emotions from our selection in each model. The model-by-model analysis excluded highly influential emotions from each respective model; thus, the models differed in which and how many emotions were included. Analysis with 59 emotions excluded emotions identified as highly influential in any model from all three models (*Bitterness*, *Disenchantment*, *Disillusion*, *Optimism* and *Terror* removed).

Table S10. Comparing the regression coefficients between the different conditions when testing for infrahumanization following harm using different analyses

| Analysis | Models being compared | *z* | *p* | *Cohen’s q* | Same result using Pearson *r*? | Same result with partial regression? |
| --- | --- | --- | --- | --- | --- | --- |
| 64 emotions | **HR & HNR** | .082 | .934 | .02 | Yes | Yes |
|  | **HR & NH** | .028 | .978 | .01 | Yes | Yes |
|  | **HNR & NH** | .11 | .913 | .02 | Yes | Yes |
| Model-by-model | **HR & HNR** | .413 | .68 | .08 | Yes | Yes |
|  | **HR & NH** | .122 | .903 | .02 | Yes | Yes |
|  | **HNR & NH** | .291 | .771 | .05 | Yes | Yes |
| 59 emotions | **HR & HNR** | .132 | .90 | .03 | Yes | Yes |
|  | **HR & NH** | .176 | .861 | .03 | Yes | Yes |
|  | **HNR & NH** | .307 | .759 | .06 | Yes | Yes |

*Notes: HR =*Harm responsible condition; *HNR* = Harm not responsible condition; *NH* = No harm condition. Results of Fisher Z transformations performed using the cocor package in R are displayed. The theoretically relevant comparison is between HR & HNR, while the comparisons between HR & NH, and HNR & NH are not theoretically relevant. Comparing Pearson correlation coefficients between emotion humanness and outgroup emotion ratings, rather than regression coefficients, yielded the same results. Comparing partial regression coefficients while controlling for emotion valence yielded the same results. No meaningful difference can be seen between the different analyses.

## Alternative Analyses when Testing for Negative Affect when Harmed.

Alternative results when deciding which highly influential emotions were excluded (if any) are displayed in Table S11, and associated main results are displayed in Table S12.

Table S11. Results of regression models using different analyses when testing for negative affect when harmed

| Analysis | Condition | *F* | *df* | *b* | *p* | *R^2^* | *r** |
| --- | --- | --- | --- | --- | --- | --- | --- |
| 64 emotions in all models | **HR** | 25.41 | 1,62 | -.161 | .001 | .29 | -.54 |
|  | **HNR** | 67.94 | 1,62 | -.277 | .001 | .52 | -.723 |
|  | **NH** | 236.7 | 1,62 | .314 | .001 | .79 | .891 |
| Model-by-model | **HR** | 52.51 | 1,59 | -.198 | .001 | .47 | -.686 |
|  | **HNR** | 116.3 | 1,59 | -.314 | .001 | .66 | -.815 |
|  | **NH** | 283.9 | 1,60 | .329 | .001 | .83 | .91 |
| 59 emotions in all models | **HR** | 44.95 | 1,57 | -.187 | .001 | .44 | -.664 |
|  | **HNR** | 104.9 | 1,57 | -.307 | .001 | .65 | -.805 |
|  | **NH** | 255 | 1,57 | .318 | .001 | .82 | .904 |

*Notes: HR =*Harm responsible condition; *HNR* = Harm not responsible condition; *NH* = No harm condition; *b* = coefficient of the predictor variable (emotion valence); r** =*Pearson correlation coefficient between emotion humanness and outgroup emotion rating. Analysis with 64 emotions in all models included all emotions from our selection in each model. The model-by-model analysis excluded highly influential emotions from each respective model; thus, the models differed in which emotions were included. Analysis with 59 emotions excluded emotions identified as highly influential in any model from all three models (*Guilt, Love, Shame, Euphoria* and *Grief* removed). No meaningful difference can be seen between the different analyses.

Table S12. Comparing the regression coefficients between the different conditions when testing for negative affect when harmed, using different analyses

| Analysis | Models being compared | *z* | *p* | *Cohen’s q* | Same result using Pearson r? | Same result with partial regression? |
| --- | --- | --- | --- | --- | --- | --- |
| 64 emotions | **HR & HNR** | 0.678 | .498 | .12 | Yes | Yes |
|  | **HR & NH** | 2.691 | .007 | .49 | Yes | Yes |
|  | **HNR & NH** | 3.368 | < .001 | .61 | Yes | Yes |
| Model-by-model | **HR & HNR** | 0.672 | .502 | .13 | Yes | Yes |
|  | **HR & NH** | 2.934 | .003 | .54 | Yes | Yes |
|  | **HNR & NH** | 3.609 | < .001 | .67 | Yes | Yes |
| 59 emotions | **HR & HNR** | 0.677 | .498 | .13 | Yes | Yes |
|  | **HR & NH** | 2.749 | .006 | .52 | Yes | Yes |
|  | **HNR & NH** | 3.426 | < .001 | .65 | Yes | Yes |

*Notes: Cond 1 =*Harm responsible condition; *Cond 2* = Harm not responsible condition; *Cond 3* = No harm condition. Results of Fisher Z transformations performed using the cocor package in R are displayed. The theoretically relevant comparisons are between Conditions 1 & 3, and 2 & 3, whereas the comparison between HR & HNR is not theoretically relevant. Comparing Pearson correlation coefficients between emotion valence and outgroup emotion ratings, rather than regression coefficients, yielded the same results. Comparing partial regression coefficients while controlling for emotion humanness yielded the same results. No meaningful differences can be seen between the different analyses.

# Equivalence testing for Studies 1 and 2

To assess whether the null effects observed in Studies 1 and 2 were robust, we conducted equivalence tests in JASP. The equivalence bounds were set to Cohen’s d = ±0.5, in line with the medium effect size thresholds specified in our power analysis. A significant result in these tests indicates that the true differences in emotion attribution ratings between the harm-responsible and harm-not-responsible groups are likely to fall within a range that can be considered practically negligible.

We also conducted Bayesian equivalence tests using the same equivalence bounds. Bayes factors (BFs) greater than 1 indicate stronger support for the equivalence hypothesis (i.e., the true effect lies within the equivalence range) over the alternative hypothesis (i.e., the effect lies outside the bounds).

The results for Study 1 and Study 2 are presented in Table S13. In Study 1, the tests revealed no evidence of meaningful group differences beyond the upper and lower bounds for any of the emotion categories (all p-values ≤ .031). The corresponding BFs provided evidence approaching moderate support for group differences falling within the equivalence range (all BFs ≥ 2.5).

Similarly, in Study 2, the tests provided support for the true effects falling within the equivalence bounds (all p-values ≤ .032; all BFs ≥ 2.5), further supporting the absence of meaningful group differences.

Table S13. Equivalence testing results for Studies 1 and 2.

| **Study** | **Emotion category** | ***Upper bound*** | | ***Lower bound*** | |  |
| --- | --- | --- | --- | --- | --- | --- |
|  |  | ***t*** | ***p*** | ***t*** | ***p*** | **BF** |
| 1 | Uniquely human positive | 4.61 | < .001 | -1.88 | .031 | 2.50 |
|  | Uniquely human negative | 2.65 | .004 | -3.83 | < .001 | 2.55 |
|  | Shared positive | 4.48 | < .001 | -2.00 | .023 | 2.51 |
|  | Shared negative | 3.23 | < .001 | -3.25 | < .001 | 2.55 |
| 2 | Uniquely human positive | 4.57 | < .001 | -1.92 | .029 | 2.50 |
|  | Uniquely human negative | 3.54 | < .001 | -2.94 | .002 | 2.55 |
|  | Shared positive | 4.62 | < .001 | -1.86 | .032 | 2.50 |
|  | Shared negative | 2.68 | .004 | -3.81 | < .001 | 2.55 |
